# Global photosynthetic capacity is optimized to the environment

**DOI:** 10.1111/ele.13210

**Published:** 2019-01-04

**Authors:** Nicholas G. Smith, Trevor F. Keenan, I. Colin Prentice, Han Wang, Ian J. Wright, Ülo Niinemets, Kristine Y. Crous, Tomas F. Domingues, Rossella Guerrieri, F. Yoko Ishida, Jens Kattge, Eric L. Kruger, Vincent Maire, Alistair Rogers, Shawn P. Serbin, Lasse Tarvainen, Henrique F. Togashi, Philip A. Townsend, Meng Wang, Lasantha K. Weerasinghe, Shuang‐Xi Zhou

**Affiliations:** ^1^ Department of Biological Sciences Texas Tech University Lubbock TX USA; ^2^ Climate and Ecosystem Sciences Division Lawrence Berkeley National Laboratory Berkeley CA USA; ^3^ Department of Environmental Science, Policy and Management UC Berkeley Berkeley CA USA; ^4^ AXA Chair of Biosphere and Climate Impacts Department of Life Sciences Imperial College London London UK; ^5^ College of Forestry Northwest A&F University Yangling China; ^6^ Department of Biological Sciences Macquarie University NSW 2109 Australia; ^7^ Department of Earth System Science Tsinghua University Beijing; ^8^ Department of Plant Physiology Institute of Agricultural and Environmental Sciences Estonian University of Life Sciences Tartu Estonia; ^9^ Hawkesbury Institute for the Environment Western Sydney University Penrith Australia; ^10^ Departamento de Biologia Faculdade de Filosofia Ciências e Letras de Ribeirão Preto ‐ University of São Paulo São Paulo Brazil; ^11^ Center for Ecological Research and Forestry Applications Universidad Autonoma de Barcelona Cerdanyola Barcelona Spain; ^12^ School of Geosciences University of Edinburgh Edinburgh UK; ^13^ Centre for Tropical Environmental and Sustainability Science College of Science and Engineering James Cook University Cairns Australia; ^14^ Max Planck Institute for Biogeochemistry Jena Germany; ^15^ German Center for Integrative Biodiversity Research Halle‐Jena‐Leipzig Leipzig Germany; ^16^ Department of Forest and Wildlife Ecology University of Wisconsin – Madison Madison Wisconsin USA; ^17^ Département des sciences de l'environnement Université du Québec à Trois Rivières Trois Rivières Canada; ^18^ Environmental and Climate Sciences Department Brookhaven National Laboratory Upton NY USA; ^19^ Department of Biological and Environmental Sciences University of Gothenburg Gothenburg Sweden; ^20^ State Environmental Protection Key Laboratory of Wetland Ecology and Vegetation Restoration Northeast Normal University Changchun China; ^21^ Research School of Biology The Australian National University Canberra Australia; ^22^ Faculty of Agriculture University of Peradeniya Peradeniya Sri Lanka; ^23^ The New Zealand Institute for Plant and Food Research Ltd Hawke's Bay New Zealand

**Keywords:** Carbon cycle, Carboxylation, coordination, ecophysiology, electron transport, Jmax, light availability, nitrogen availability, temperature, V_cmax_

## Abstract

Earth system models (ESMs) use photosynthetic capacity, indexed by the maximum Rubisco carboxylation rate (*V*_cmax_), to simulate carbon assimilation and typically rely on empirical estimates, including an assumed dependence on leaf nitrogen determined from soil fertility. In contrast, new theory, based on biochemical coordination and co‐optimization of carboxylation and water costs for photosynthesis, suggests that optimal *V*_cmax_ can be predicted from climate alone, irrespective of soil fertility. Here, we develop this theory and find it captures 64% of observed variability in a global, field‐measured *V*_cmax_ dataset for C_3_ plants. Soil fertility indices explained substantially less variation (32%). These results indicate that environmentally regulated biophysical constraints and light availability are the first‐order drivers of global photosynthetic capacity. Through acclimation and adaptation, plants efficiently utilize resources at the leaf level, thus maximizing potential resource use for growth and reproduction. Our theory offers a robust strategy for dynamically predicting photosynthetic capacity in ESMs.

## Introduction

Ecosystem and Earth system models are highly sensitive to the representation of photosynthetic processes (Rogers *et al*. 2017a). In the majority of these models, C_3_ photosynthesis is simulated using well‐established biochemical theory (Farquhar *et al*. 1980). The applicability of the theory relies on knowledge of photosynthetic capacity, which varies both among species and over time and space, in response to environmental conditions (Ali *et al*. 2015; Smith & Dukes 2018).

Photosynthetic capacity is also known to correlate with leaf nitrogen (N) across plant types as a result of the N used to build photosynthetic machinery (Walker *et al*. 2014). Many global models use these empirical relationships to predict the maximum rate of Rubisco carboxylation (*V*_cmax_; μmol m^−2^ s^−1^), a primary determinant of photosynthetic capacity (Rogers 2014). This approach inherently assumes that variation in *V*_cmax_ is driven by variation in N allocated to leaves, which is itself prescribed or calculated from N availability in soils. This leads to a positive relationship between *V*_cmax_ and soil N availability. This approach was shown to perform well in a comparison of several model formulations (Walker *et al*. 2017). However, there are several important limitations to the N‐supply approach for predicting *V*_cmax_. First, observed relationships between field‐measured *V*_cmax_ and leaf N per leaf area (*N*_a_) are often only weak (e.g. *r* ^2^ = 0.3; Niinemets *et al*. 2009). Second, an increase in *V*_cmax_ per leaf *N*_a_ at lower soil N availability (Ainsworth & Rogers 2007; Kattge *et al*. 2009; Maire *et al*. 2012) suggests that high *V*_cmax_ can be achieved under low soil N. Third, the N‐supply approach is necessarily empirical, yet it is only with mechanistic models that we stand to reliably predict responses to future, novel conditions.

Photosynthetic coordination theory provides an approach to predict dynamic responses of photosynthetic capacity to environmental constraints. Originally proposed by Von Caemmerer & Farquhar (1981) and further developed by Chen *et al*. (1993), Maire *et al*. (2012) and Wang *et al*. (2017c), it states that photosynthesis tends to be equally limited by electron transport and carboxylation under average environmental conditions. Notably, while this implicitly assumes dynamic nutrient partitioning within leaves, it does not assume any nutrient availability constraint on carboxylation rates, electron transport rates or the partitioning of nitrogen between the two. While this response may be possible under any given amount of N availability, here, we present a ‘strong’ form of the coordination theory, which assumes that plants are able to acquire the N necessary to build leaves that can photosynthesize at the fastest possible rate given light availability and biophysical constraints, for example, through increased belowground allocation (Drake *et al*. 2011; Terrer *et al*. 2016). This is quite different, in formulation and consequences, from other interpretations that focus on the partitioning of a fixed amount of N to *V*_cmax_ versus *J*_max_ (e.g. Ali *et al*. 2016).

In this study, we tested a theoretical framework for predicting *V*_cmax_ from first principles at the global scale. Building on work from Dong *et al*. (2017), Wang *et al*. (2017b) and Togashi *et al*. (2018b), our approach works by combining photosynthetic coordination theory with ‘least‐cost’ theory for understanding investments in carboxylation and water transport capacities for photosynthesis (Wright *et al*. 2003; Prentice *et al*. 2014). The least‐cost hypothesis posits that these investments are co‐optimized in relation to environmental properties such that a given photosynthetic rate is achieved at the lowest total cost (i.e. respiration). From this principle, one can predict the optimal CO_2_ drawdown during photosynthesis (i.e. intercellular to atmospheric CO_2_ or *C*_i_:*C*_a_) as a function of site temperature, vapour pressure deficit and atmospheric pressure (Prentice *et al*. 2014; Wang *et al*. 2017c). By drawing together the least‐cost and coordination theory, an important step forward is possible: as outlined in the Methods, *V*_cmax_ can in theory be predicted as a function of light availability (*I*), temperature (*T*), vapour pressure deficit (*D*) and atmospheric pressure (as indexed by elevation, *z*).

Here, we test this proposition, using a dataset of 3672 values of *V*_cmax_ from 201 sites from across the globe. First, we tested our quantitative predictions for individual effects of *I*,* T*,* D* and *z* on *V*_cmax_ and compared model‐predicted *V*_cmax_ to observed *V*_cmax_ values. Second, we examined the sensitivity of our *V*_cmax_ predictions to *I*,* T*,* D* and *z* as well as leaf traits not included in the model, namely leaf nitrogen per leaf area (*N*_a_) and leaf mass per area (*LMA*). Finally, we used six soil indices to explore the relative influence of soil N and water supply and environmental constraints on *V*_cmax_. Using these data, we indirectly tested the proposition that leaf N concentrations more strongly reflect ‘demand’ for N (the need to support a given *V*_cmax_, itself optimized to climate) rather than ‘supply’ of N (from the soil).

## Materials and methods

### Observational *V*_cmax_ dataset

An observational dataset of *V*_cmax_ values was built by combining independent data reported to be from top canopy, natural vegetation from Bahar *et al*. (2017), Carswell *et al*. (2000), De Kauwe *et al*. (2016), Domingues *et al*. (2010, 2015), Ellsworth & Crous (2016), Keenan & Niinemets (2016), Maire *et al*. (2015), Meir *et al*. (2002), Niinemets *et al*. (2015), Rogers *et al*. (2017b), Serbin *et al*. (2015), Smith & Dukes (2017a), Tarvainen *et al*. (2013), Togashi *et al*. (2018a,b), the TRY plant trait database (Kattge *et al*. 2011), Wang *et al*. (2017a) and Wohlfahrt *et al*. (1999) (Figure S1 and S2). *V*_cmax_ values in the dataset were derived from either net photosynthesis (*A*_net_) to intercellular CO_2_ (*C*_i_; 56% of the total dataset) curves or from point measurements of *A*_net_ and *C*_i_ using the one‐point method (44%; method presented in De Kauwe *et al*. (2016); see discussion of the limitations of this method in the Supplementary Information). The dataset includes latitude, longitude and leaf temperature at the time of measurement for each point and, for a subset of the data, leaf nitrogen content per unit leaf area (*N*_a_; gN m^−2^; 57% of the dataset) and leaf mass per unit leaf area (*LMA*; g m^−2^; 60% of the dataset). Latitude and longitude were used to extract effective growing season mean temperature (*T*_g_; °C), atmospheric vapour pressure deficit (*D*_g_; Pa) and incoming photosynthetically active radiation (*I*_g_; μmol m^−2^ s^−1^) for each site from monthly, 1901–2015, 0.5° resolution data provided by the Climatic Research Unit (CRU TS3.24.01) (Harris *et al*. 2014). Growing season was operationally defined as months with mean temperatures greater than 0 °C. The elevation (*z*; m) at each site at 0.5° resolution was obtained from the WFDEI meteorological forcing dataset (Weedon *et al*. 2014). The ratio of actual evapotranspiration to equilibrium evapotranspiration (Priestley‐Taylor coefficient, α), which represents the plant‐available surface moisture, was calculated at each 0.5° resolution site using the SPLASH model run at a monthly timescale (Davis *et al*. 2017). Soil cation exchange capacity (CEC; cmol_c_ kg^−1^), soil pH, soil C:N ratio, soil silt content (%) and soil clay content (%) at 0–40 cm depth were extracted from 1 km global data provided by ISRIC SoilGrids database (http://www.soilgrids.org). These soil data were available for 97% of the total dataset.

### Theoretical model of *V*_cmax_

The theoretical model of *V*_cmax_ was developed from the theory presented by Wang *et al*. (2017c) and Dong *et al*. (2017) by combining the coordination theory of photosynthesis (Maire *et al*. 2012) with the least‐cost hypothesis (Wright *et al*. 2003; Prentice *et al*. 2014). The combination of the two theories is done by calculating an optimal intercellular CO_2_ concentration under average environmental conditions ($C_{i}^{\prime }$), which is then used to calculate optimal *V*_cmax_ under the same conditions ($V_{\mathrm{cmax}}^{\prime }$). These calculations were made using light, temperature, vapour pressure deficit, elevation and atmospheric CO_2_ as inputs. We first present the formulations for calculating the $C_{i}^{\prime }$ values used in the optimal $V_{\mathrm{cmax}}^{\prime }$ prediction following Prentice *et al*. (2014). We then describe how we use coordination theory to predict optimal $V_{\mathrm{cmax}}^{\prime }$ (equation (20) below).

### Optimal C_i_ calculation

**Figure 1:**
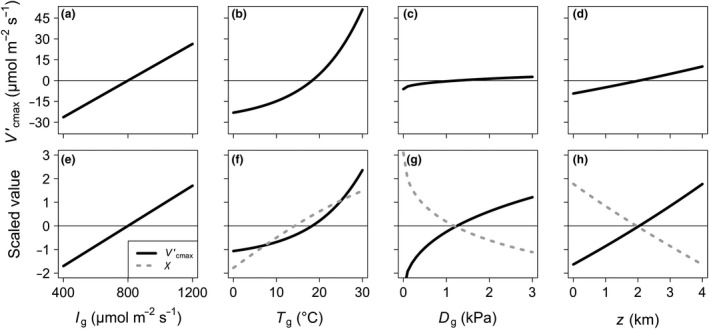
Sensitivity of the theoretical model to environmental drivers. Sensitivity of the theoretical maximum rate of Rubisco carboxylation ($V_{\mathrm{cmax}}^{\prime }$; black, solid lines) and ratio of intercellular to atmospheric CO_2_ concentration (χ*;* grey dotted lines, panels f, g and h) to the main environmental parameters within the model: growing season mean for irradiance (*I*_g_, panels a and e), air temperature (*T*_g_, panels b and f) and vapour pressure deficit (*D*_g_, panels c and g), as well as elevation (*z*, panels d and h). In panels a, b, c and d, $V_{\mathrm{cmax}}^{\prime }$ values were mean centred to aid in comparison across environmental parameters. In panels e, f, g and h, values were mean centred and scaled (divided by the standard deviation) to aid comparison of $V_{\mathrm{cmax}}^{\prime }$and χ sensitivities. Sensitivity analyses were done while keeping all other environmental variables at standard levels: *I*_g_ **= 800 μmol m^−2^ s^−1^, *T*_g_ **= 25 °C, *D*_g_ **= 1 kPa, z = 0 km. Note: χ is insensitive to *I*_g_, and as such, no dashed grey line was plotted_._

The optimal intercellular CO_2_ concentration under average environmental conditions ($C_{i}^{\prime }$; Pa) was calculated using a theoretical derivation of the optimal ratio (χ) of $C_{i}^{\prime }$ to atmospheric CO_2_ partial pressure (*C*_*a*_; Pa), based on least‐cost theory from Prentice *et al*. (2014):

(1) $$\chi =\frac{\Gamma^{*}}{C_{a}}+\left(1-\frac{\Gamma^{*}}{C_{a}}\right)\frac{\xi }{\xi +\sqrt{D_{g}}}$$

where

(2) $$\xi =\sqrt{\beta \frac{K+\Gamma^{*}}{1.6\eta^{*}}}$$

where ξ defines the sensitivity of χ to *D*_g_ and is related to the carbon cost of water (Medlyn *et al*. 2011; Prentice *et al*. 2014), $\Gamma^{*}$ (Pa) is the CO_2_ compensation point in the absence of mitochondrial respiration, and *K* (Pa) is as follows:

(3) $$K=K_{c}\left(1+\frac{O_{i}}{K_{o}}\right)$$

where *K*_c_ (Pa) and *K*_o_ (Pa) are Michaelis–Menten coefficients of Rubisco activity for CO_2_ and O_2_, respectively, and *O*_i_ (Pa) is the intercellular O_2_ concentration. A consideration of O_2_ concentrations is included to account for declines in carboxylation that occur as a result of Rubisco oxygenation. Values of *K* and $\Gamma^{*}$ are temperature dependent and were calculated using the equations and parameters of Bernacchi *et al*. (2001) using *T*_g_. The term β (unitless) in equation (2) is the ratio (*b/a*) of dimensionless cost factors describing the carbon cost of maintaining photosynthetic proteins to support assimilation at a given rate under normal daytime conditions (*b*) and the carbon cost of maintaining a transpiration stream to support assimilation at the same rate (*a*) (Prentice *et al*. 2014). We used a constant β, estimated as 146, calculated under standard conditions (*T*_*g*_ = 25 °C, *D*_*g*_ = 1 kPa, *z *=* *0) from χ values derived from leaf stable carbon isotope data (Cornwell 2017) and equations 1 and 2, as in Wang *et al*. (2017c).$\eta^{*}$ is the viscosity of water relative to its value at 25 °C, calculated using temperature and elevation as in Huber *et al*. (2009). In cases where *C*_a_ was unknown, we used the year of measurement to estimate *C*_a_ from global estimates used by the NASA GISS model, which utilizes a combination of measurements and modelling techniques to estimate a global average *C*_a_ (https://data.giss.nasa.gov/modelforce/ghgases/Fig 1A.ext.txt).

### Optimal *V*_cmax_ calculation

We calculated the optimal maximum rate of Rubisco carboxylation under average environmental conditions ($V_{\mathrm{cmax}}^{\prime }$) by assuming that, optimally, plants will coordinate the allocation of resources to photosynthesis such that under typical environmental conditions:

(4) $$A_{c}=A_{j}$$

where *A*_c_ (μmol m^−2^ s^−1^) is the photosynthetic rate limited by the maximum rate of Rubisco carboxylation (*V*_cmax_; μmol m^−2^ s^−1^):

(5) $$A_{c}=V_{\mathrm{cmax}}m_{c}$$

where

(6) $$m_{c}=\frac{C_{i}^{\prime }-\Gamma^{*}}{C_{i}^{\prime }+K}$$

where $C_{i}^{\prime }$(Pa), $\Gamma^{*}$ (Pa) and *K* (Pa) are calculated as in the previous section.

*A*_j_ (μmol m^−2^ s^−1^) is the photosynthetic rate limited by the electron transport rate for the regeneration of ribulose‐1,5,‐bisphosphate (*RuBP*;* J*; μmol m^−2^ s^−1^):

(7) $$A_{j}=\left(\frac{J}{4}\right)m$$

where

(8) $$m=\frac{C_{i}^{\prime }-\Gamma^{*}}{C_{i}^{\prime }+2\Gamma^{*}}$$

*J* is a saturating function of irradiance, converging on *J*_max_ (μmol m^−2^ s^−1^) at high levels:

(9) $$\theta J^{2}-\left(\varphi I+J_{\max}\right)J+\varphi IJ_{\max}=0$$

where *I* is the incident photosynthetically active photon flux density (μmol m^−2^ s^−1^), θ (unitless) is the curvature of the light response curve, and φ is the realized quantum yield of photosynthetic electron transport (mol mol^−1^) (Farquhar & Wong 1984). We adopted a value of φ of 0.257 mol mol^−1^, which yielded a slope between the measured and predicted $V_{\mathrm{cmax}}^{\prime }$ values near 1. This φ value is within the range of values observed by independent, leaf‐level studies (0.26 in soya bean (June 2005), 0.23 in soya bean (Harley *et al*. 1985), 0.28 in *Eucalyptus pauciflora* (Kirschbaum & Farquhar 1987), and 0.26 in a seven‐species analysis (Ehleringer & Björkman 1977)). The curvature term, θ, is related to the distribution of light intensity relative to the distribution of photosynthetic capacity, assumed to be 0.85, consistent with observations (June 2005). Eqn (9) can be substituted into eqn (7) to yield

(10) $$A_{j}=\left(\frac{m}{4}\right)\frac{\varphi I+J_{\max}\pm \sqrt{{\left(\varphi I+J_{\max}\right)}^{2}-4\theta \varphi IJ_{\max}}}{2\theta }$$

from which the smaller root is used to derive *A*_j_.

To derive optimal *J*_max_, we assumed that *A*_j_ changes in proportion to *J*_max_, as proposed by Farquhar (1989). As such, we took the derivative of *A*_j_ (Eqn (10)) with respect to *J*_*max*_ and equated this to *c*:

(11) $$c=\frac{\partial A_{j}}{\partial J_{\max}}$$

*c* is then given by

(12) $$c=\left(\frac{m}{4}\right)\frac{\partial }{\partial J_{\max}}\left(\frac{\varphi I+J_{\max}-\sqrt{{\left(\varphi I+J_{\max}\right)}^{2}-4\theta \varphi IJ_{\max}}}{2\theta }\right)$$

which simplifies to

(13) $$c=\frac{m}{8\theta }\left(1-\frac{\partial }{\partial J_{\max}}\sqrt{{\left(\varphi I+J_{\max}\right)}^{2}-4\theta \varphi IJ_{\max}}\right)$$

which can be solved as

(14) $$c=\frac{m}{8\theta }\left(1-\frac{\varphi I+J_{\max}-2\theta \varphi I}{\sqrt{{\left(\varphi I+J_{\max}\right)}^{2}-4\theta \varphi IJ_{\max}}}\right)$$

Equation (14) can be rearranged to:

(15) $$J_{\max}=\varphi I\varpi$$

where

(16) $$\varpi =-\left(1-2\theta \right)+\sqrt{\left(1-\theta \right)\left(\frac{1}{\frac{4c}{m}\left(1-\theta \frac{4c}{m}\right)}-4\theta \right)}$$

For the calculation of ϖ, *c* was assumed to be non‐varying and derived as 0.053 under standard conditions (see Supplementary Information). We then inserted the solution for *J*_max_ into eqn (10) and solved for *A*_j_:

(17) $$A_{j}=\frac{\varphi Im\varpi^{*}}{8\theta }$$

where

(18) $$\varpi^{*}=1+\varpi -\sqrt{{\left(1+\varpi \right)}^{2}-4\theta \varpi }$$

Finally, eqns 5 and 17 were used to replace *A*_c_ and *A*_j_ in equation 4 and solve for an intermediate rate of *V*_cmax_, which we term *V*_cmax_^*^:

(19) $${V_{\mathrm{cmax}}}^{*}=\varphi I\left(\frac{m}{m_{c}}\right)\left(\frac{\varpi^{*}}{8\theta }\right)$$

Equation 19 incorporates the temperature response of *m* and *m*_c_. However, *V*_cmax_ itself (i.e. the saturation point of the Michaelis–Menten curve) is also sensitive to temperature. As such, we used a formulation from Kattge & Knorr (2007) to incorporate this temperature response, which yielded $V_{\mathrm{cmax}\left[\mathrm{pred}\right]}^{\prime }$ or predicted *V*_cmax_ acclimated to varying environmental conditions):

(20) $$V_{\mathrm{cmax}\left[\mathrm{pred}\right]}^{\prime }=\left({V_{\mathrm{cmax}}}^{*}\right)e^{\frac{H_{a}\left(T_{g}-T_{o}\right)}{RT_{g}T_{o}}}\frac{1+e^{\frac{T_{o}\left(\Delta S\right)-H_{d}}{RT_{o}}}}{1+e^{\frac{T_{g}\left(\Delta S\right)-H_{d}}{RT_{g}}}}$$

where *H*_d_ is the deactivation energy (200 000 J mol^−1^), *H*_a_ is the activation energy (71,513 J mol^−1^), *R* is the universal gas constant (8.314 J mol^−1^ K^−1^), ∆*S* is an entropy term (J mol^−1^ K^−1^), *T*_g_ is the growing season temperature in K, and *T*_o_ is the optimum temperature in K, assumed to be the temperature at which *V*_cmax_^*^ is operating. *T*_o_ was estimated based on its relationship to growth temperature (Kattge & Knorr 2007):

(21) $$T_{o}=177.884+0.44T_{g}$$

∆*S* was calculated based on a linear relationship with *T*_g_ from Kattge & Knorr (2007), with a slope of −1.07 J mol^−1^ K^−1^ and intercept of 668.39 J mol^−1^ K^−1^ (Kattge & Knorr 2007).

In addition to $C_{i}^{\prime }$, the resulting theoretical prediction of optimal *V*_cmax_ (Eq. 20) requires only two free parameters: θ (unitless), the curvature of the light response curve, and φ, the quantum yield of photosynthetic electron transport (mol mol^−1^).

### Model‐data comparison

To perform the model‐data comparison, we standardized each observed *V*_cmax_ value ($V_{cmax\left[\mathrm{meas}\right]}$) to its *T*_g_ (i.e. $V_{\mathrm{cmax}\left[\mathrm{obs}\right]}^{\prime }$) using temperature response formulations from Kattge & Knorr (2007):

(22) $$V_{\mathrm{cmax}\left[\mathrm{obs}\right]}^{\prime }=V_{\mathrm{cmax}\left[\mathrm{meas}\right]}e^{\frac{H_{a}\left(T_{g}-T_{\mathrm{meas}}\right)}{RT_{g}T_{\mathrm{meas}}}}\frac{1+e^{\frac{T_{\mathrm{meas}}\left(\Delta S\right)-H_{d}}{RT_{\mathrm{meas}}}}}{1+e^{\frac{T_{g}\left(\Delta S\right)-H_{d}}{RT_{g}}}}$$

where *T*_meas_ is the leaf temperature at which the measurement was taken (K), *V*_cmax[meas]_ is the measured *V*_cmax_, and ∆*S* was calculated as in eqn. 20 from *T*_g_ following Kattge & Knorr (2007). Next, we used the theoretical model described above to predict *V*_cmax_ values at the *T*_g_ for each observation (i.e. $V_{\mathrm{cmax}\left[\mathrm{obs}\right]}^{\prime }$). We then aggregated the predicted and $V_{\mathrm{cmax}\left[\mathrm{obs}\right]}^{\prime }$ values by latitude and longitude at a resolution of 0.5 °C to match the climatological data. Finally, we used Model II Reduced Major Axis slope‐fitting (R package ‘lmodel2′ (Legendre 2014)) to compare predicted and observed rates of $V_{\mathrm{cmax}}^{\prime }$ at each site. To examine the ability of our model to simulate the ratio of $J_{\max}^{\prime }$ to $V_{\mathrm{cmax}}^{\prime }$ ($J_{\max}^{\prime }$/$V_{\mathrm{cmax}}^{\prime }$), we ran a similar comparison of predicted and observed $J_{\max}^{\prime }$/$V_{\mathrm{cmax}}^{\prime }$ at each of the 90 sites where $J_{\max\left[\mathrm{obs}\right]}^{\prime }$ data were available. Note, that due to the similarity between Eqns. 20 and 22 necessarily applied to predicted and observed data for comparison, we explored the potential for a spurious correlation between modelled and observed data due to a common element (Chayes 1971) (Supplementary Information). Additionally, because some *V*_cmax_ values in the observational dataset were derived using the one‐point method (method presented in De Kauwe *et al*. 2016), we ran a similar model‐data comparison as above using only data derived using *A*_net_‐*C*_i_ curves (Supplementary Information).

Following direct comparison, we calculated the model bias (*B*) in $V_{\mathrm{cmax}}^{\prime }$ predictions at each site as

(23) $$B=\frac{V_{\mathrm{cmax}\left[\mathrm{pred}\right]}^{\prime }-V_{\mathrm{cmax}\left[\mathrm{obs}\right]}^{\prime }}{V_{\mathrm{cmax}\left[\mathrm{obs}\right]}^{\prime }}*100$$

We then explored *B* as a function of the primary environmental drivers in the model, *T*_g_, *I*_g_, *D*_g_ and *z*, as well as secondary environmental variables soil cation exchange capacity, soil pH, soil C:N ratio, soil silt content, soil clay content, a soil water content index (α), leaf mass per area (*LMA*) and leaf nitrogen content (*N*_a_) using multiple linear regression. A single regression model was first fit using the four primary drivers. Following this, a second model was fit that included the four primary drivers and each of the six soil variables, which were available for 193 of 201 sites (97%).

Two additional models were fit that included all primary drivers and one of *LMA* or *N*_a_, which were available for 112 (56%) and 98 (49%) of 201 sites, respectively. All analyses were performed in R version 3.5.0.

As a further examination of the influence of soil variables on $V_{\mathrm{cmax}\left[\mathrm{obs}\right]}^{\prime }$, we fit three separate models using the 193 sites for which soil data were available. The first model, similar to above, only included $V_{\mathrm{cmax}\left[\mathrm{pred}\right]}^{\prime }$. The second model only included the six soil variables: soil cation exchange capacity, soil pH, soil C:N ratio, soil silt content, soil clay content and α. The third model included both $V_{\mathrm{cmax}\left[\mathrm{pred}\right]}^{\prime }$ and all six soil variables. The three models were compared using Akaike information criteria (AIC). We also performed a similar comparison using leaf *N*_a_ values for the 98 sites that had *N*_a_ data. For comparisons of models with and without soil variables, each model was fit using only the 193 sites where soil data were available. Similarly, for comparisons of models with and without *N*_a_, each model was fit using only the 98 sites where *N*_a_ data were available. This ensured that model comparisons were done using identical datasets. For all models, we visually examined residual plots following model fitting to ensure that necessary assumptions for model comparisons were met (Zuur *et al*. 2009). We also calculated the variance inflation factor (*VIF*) for each model predictor to assess the degree of collinearity. In all cases, *VIF* values were less than 5 and, in the case of all discussed significant predictors (i.e. *P *<* *0.05), values were less than 3, indicating that collinearity did not have a large impact on our interpretations (Zuur *et al*. 2009).

### Comparison to CANTRIP database

To examine the potential influence of canopy position on our model‐data comparison, we examined a subset of the $V_{\mathrm{cmax}\left[\mathrm{obs}\right]}^{\prime }$values in the dataset (CANTRIP) (Keenan & Niinemets 2016) that were standardized to top of the canopy light values (*Q*_*int*_ = 40 mol m^−2^ d^−1^). These values were determined using individual canopy scaling relationships, which were applied to 109 individual plant canopies (Niinemets *et al*. 2015). Separate model‐data comparisons, as described above, were performed for the full dataset without the CANTRIP data and with only the CANTRIP data. We used Student's *t*‐test to examine whether the difference between modelled and observed data differed between the non‐CANTRIP and the CANTRIP data. Both the CANTRIP and non‐CANTRIP datasets were normally distributed and had similar standard deviations.

## Results

### Predicted response of optimal $V_{\mathrm{cmax}}^{\prime }$ to environmental drivers

In response to increased light availability, our model predicted a positive, linear response of optimal $V_{\mathrm{cmax}}^{\prime }$ (i.e. $V_{\mathrm{cmax}}^{\prime }$). This effect was driven by increases in electron transport under increased light, which led to a necessary increase in $V_{\mathrm{cmax}}^{\prime }$ for carboxylation rate‐limited photosynthesis to match electron transport rate‐limited photosynthesis. Similarly, our model predicted a nonlinear increase in $V_{\mathrm{cmax}}^{\prime }$ with temperature (Fig. 1). This was the result of an increase in electron transport with temperature as well as an increased affinity of Rubisco for O_2_, which also caused an increase in χ. As a consequence, the predicted ratio of $J_{\max}^{\prime }$ to $V_{\mathrm{cmax}}^{\prime }$ decreased with increasing temperatures (Figure S5). The model predicted slight increases in $V_{\mathrm{cmax}}^{\prime }$ with increased vapour pressure deficit and elevation due to reduced stomatal conductance (Fig. 1).

### Model‐data comparison

**Figure 2:**
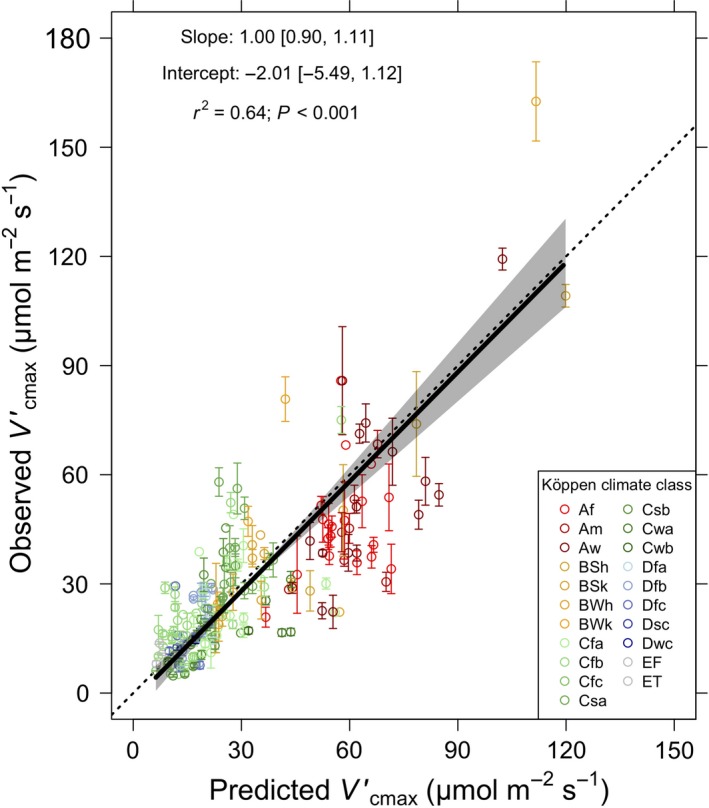
Comparison of observed to optimal $V_{\mathrm{cmax}}^{\prime }$. Observed mean maximum rate of Rubisco carboxylation ($V_{\mathrm{cmax}}^{\prime }$) at 201 global sites plotted against the predicted $V_{\mathrm{cmax}}^{\prime }$value at that site from the theoretical model. Sites are coloured by Köppen climate classification. Tropical (first letter A), arid (first letter B), temperate (first letter C), boreal (first letter D) and polar (first letter E) regions are represented by red, yellow, green, blue and grey colours. Error bars represent standard errors of the mean. The solid black line is the best fit line from the reduced major axis regression. The grey‐shaded area represents a 95% confidence interval. The dotted black line is a 1:1 line. Köppen climate classification key: Af= tropical rainforest, Am= tropical monsoon, Aw= tropical wet savannah, BSh= hot arid steppe, BSk= cold arid steppe, BWh= hot arid desert, BWk= cold arid desert, Cfa= temperate hot summer without dry season, Cfb= temperate warm summer without dry season, Cfc= temperate cold summer without dry season, Csa= temperate hot summer with dry summer, Csb= temperate warm summer with dry summer, Cwa= temperate hot summer with dry winter, Cwb= temperate warm summer with dry winter, Dfa= boreal hot summer without dry season, Dfb= boreal warm summer without dry season, Dfc= boreal cold summer without dry season, Dsc= boreal cold summer with dry summer, Dwc= boreal cold summer with dry winter, EF= eternal winter, ET= tundra. A version of this figure with individual points can be found in the Supplementary Information (Figure S8).

When compared to the global database, our theoretical model captured 64% of the total variation in $V_{\mathrm{cmax}\left[\mathrm{obs}\right]}^{\prime }$ values (Fig. 2). After tuning the model to have a slope near 1, the intercept of the relationship between observed and predicted values had a 95% confidence interval (CI) that bracketed 0 (mean = −2.01, 95% CI: ‐5.49, 1.12). The model performed similarly well using only data derived from *A*_net_‐*C*_i_ curves (r^2^ = 0.68; Supplementary Information). Our theoretical model was also able to capture 61% of the variation in $J_{\max\left[\mathrm{obs}\right]}^{\prime }$/$V_{\mathrm{cmax}\left[\mathrm{obs}\right]}^{\prime }$ at the 90 sites that contained $J_{\max\left[\mathrm{obs}\right]}^{\prime }$ data (Figure S3). The slope and intercept of the relationship between observed and predicted $J_{\max\left[\mathrm{obs}\right]}^{\prime }$/$V_{\mathrm{cmax}\left[\mathrm{obs}\right]}^{\prime }$ values had 95% confidence intervals (CI) that bracket 1 and 0, respectively (slope = 0.94, 95% CI: 0.79, 1.12; intercept =−0.44, 95% CI: −0.99, 0.02). In both cases, there was a slight overprediction of values on average across sites (Fig. 2 and Figure S3).

### Model biases – environmental drivers

**Figure 3:**
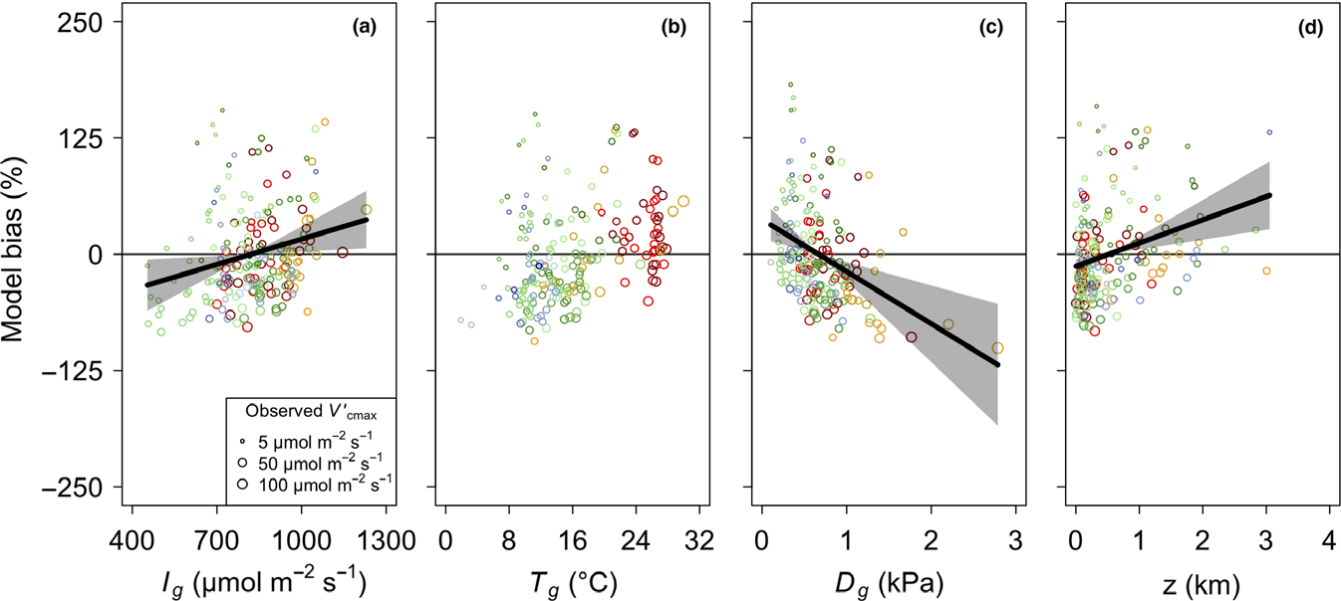
Partial residuals of the observed bias (%) in maximum rate of Rubisco carboxylation ($V_{\mathrm{cmax}}^{\prime }$) predicted by the theoretical model at each of the 201 sites plotted against growing season light (*I*_g_), growing season temperature (*T*_g_), growing season leaf‐to‐air vapour pressure deficit (*D*_g_), and elevation (*z*) (grey circles). Model bias was defined as $\frac{V_{\mathrm{cmax}\left[\mathrm{pred}\right]}^{\prime }-V_{\mathrm{cmax}\left[\mathrm{obs}\right]}^{\prime }}{V_{\mathrm{cmax}\left[\mathrm{obs}\right]}^{\prime }}*100$, where $V_{\mathrm{cmax}\left[\mathrm{pred}\right]}^{\prime }$ is the predicted optimal $V_{\mathrm{cmax}}^{\prime }$ and $V_{\mathrm{cmax}\left[\mathrm{obs}\right]}^{\prime }$ is the observed $V_{\mathrm{cmax}}^{\prime }$. Data points are sized logarithmically by $V_{\mathrm{cmax}\left[\mathrm{obs}\right]}^{\prime }$. Lines indicate the modelled response from the multiple linear regression models. Shading indicates 95% confidence intervals for regression lines. Only significant trends (*P* < 0.05) are shown. Colours are as in Figure 2.

Our theoretical model showed a positive bias with growing season mean irradiance (Fig. 3 and Table S1; *F*_1,196_ = 11.54, *P* < 0.01). This was driven by an overprediction in wet, tropical regions (Fig. 2), potentially due to an overestimation of incoming light in dense tropical forests. To explore whether this was due to an overestimation of light availability, we compared the accuracy of our theory using high‐light $V_{\mathrm{cmax}\left[\mathrm{obs}\right]}^{\prime }$ estimates from the CANTRIP database (Keenan & Niinemets 2016), which are not influenced by canopy shading. The model tended to underpredict the CANTRIP $V_{\mathrm{cmax}\left[\mathrm{obs}\right]}^{\prime }$ rates to a greater degree than non‐CANTRIP rates (Figure S4; *t*_76.2_=‐2.912, *P *<* *0.01). This result suggests that some data in the observational dataset may have been collected from leaves growing under non‐maximum light conditions.

The warmest and driest environments in our dataset (*D*_g_ > 1.5 kPa) showed the greatest underestimation of $V_{\mathrm{cmax}\left[\mathrm{obs}\right]}^{\prime }$, leading to a slight negative bias overall (Fig. 3; *F*_1,196_ = 7.66, *P *<* *0.01). Our model also tended to overpredict $V_{\mathrm{cmax}\left[\mathrm{obs}\right]}^{\prime }$ at elevations above c. 1500 m (Fig. 3), which led to a significant positive bias in our model with elevation (*F*_1,196_ = 11.62, *P* < 0.01). There was no systematic bias in our model related to *T*_g_ (Fig. 3; *F*_1,196_ = 2.19, *P* = 0.14).

### Model biases – leaf traits

**Figure 4:**
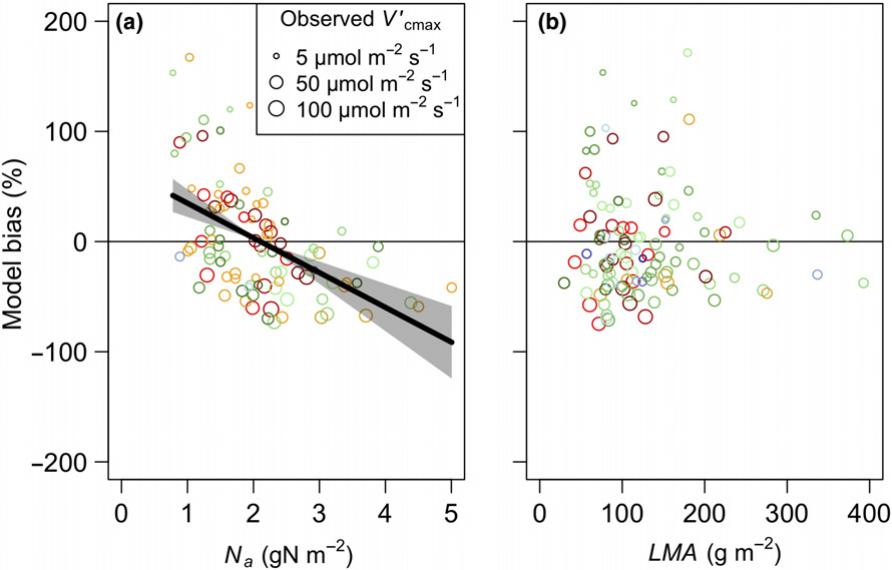
Partial residuals of the observed bias (%) in maximum rate of Rubisco carboxylation ($V_{\mathrm{cmax}}^{\prime }$) predicted by the theoretical model by site plotted against leaf nitrogen per leaf area (*N*_a_; *n* = 98) and leaf mass per leaf area (LMA;* n* = 112) (grey circles). Model bias was defined as $\frac{V_{\mathrm{cmax}\left[\mathrm{pred}\right]}^{\prime }-V_{\mathrm{cmax}\left[\mathrm{obs}\right]}^{\prime }}{V_{\mathrm{cmax}\left[\mathrm{obs}\right]}^{\prime }}*100$, where $V_{\mathrm{cmax}\left[\mathrm{pred}\right]}^{\prime }$ is the predicted optimal $V_{\mathrm{cmax}}^{\prime }$ and $V_{\mathrm{cmax}\left[\mathrm{obs}\right]}^{\prime }$ is the observed $V_{\mathrm{cmax}}^{\prime }$. Data points are sized logarithmically by $V_{\mathrm{cmax}\left[\mathrm{obs}\right]}^{\prime }$. Lines indicate the modelled response from the multiple linear regression models. Shading indicates 95% confidence intervals for regression lines. Only significant trends (P < 0.05) are shown. Colours are as in Figure 2.

When evaluated across variation in *N*_a_ our theory showed a negative bias, indicating an overestimation of $V_{cmax\left[\mathrm{obs}\right]}^{\prime }$ among low *N*_a_ sites and underestimation at high *N*_a_ sites (Fig. 4 and Table S2; *F*_1,92_ = 29.67, *P* < 0.01). To explore the relative impact of *N*_a_ versus climate and environmental variables driving the optimality model, we fit three linear regression models predicting $V_{\mathrm{cmax}\left[\mathrm{obs}\right]}^{\prime }$: one with $V_{\mathrm{cmax}\left[\mathrm{pred}\right]}^{\prime }$ *,* a second with *N*_a_, and a third with $V_{\mathrm{cmax}\left[\mathrm{pred}\right]}^{\prime }$ and *N*_a_, each using the same subset of the dataset where *N*_a_ was reported (*n* = 98 sites). The fit of the model that included both $V_{\mathrm{cmax}\left[\mathrm{pred}\right]}^{\prime }$ and *N*_a_ (AIC = 724.5, *r* ^2^ = 0.67) was slightly better than the model that included just $V_{\mathrm{cmax}\left[\mathrm{pred}\right]}^{\prime }$ (AIC = 741.7, *r* ^2^ = 0.60) and substantially better than the model that included *N*_a_ (AIC = 828.4, *r* ^2^ = 0.03), suggesting that, while *N*_a_ did add significant predictive value, environmental constraints and light availability (indexed by $V_{\mathrm{cmax}\left[\mathrm{pred}\right]}^{\prime }$) are the dominant drivers of photosynthetic capacity. Our theory showed no bias in response to LMA (Fig. 4 and Table S3; *F*_1,106_ = 0.09, *P* = 0.76).

### Model biases – soil characteristics

**Figure 5:**
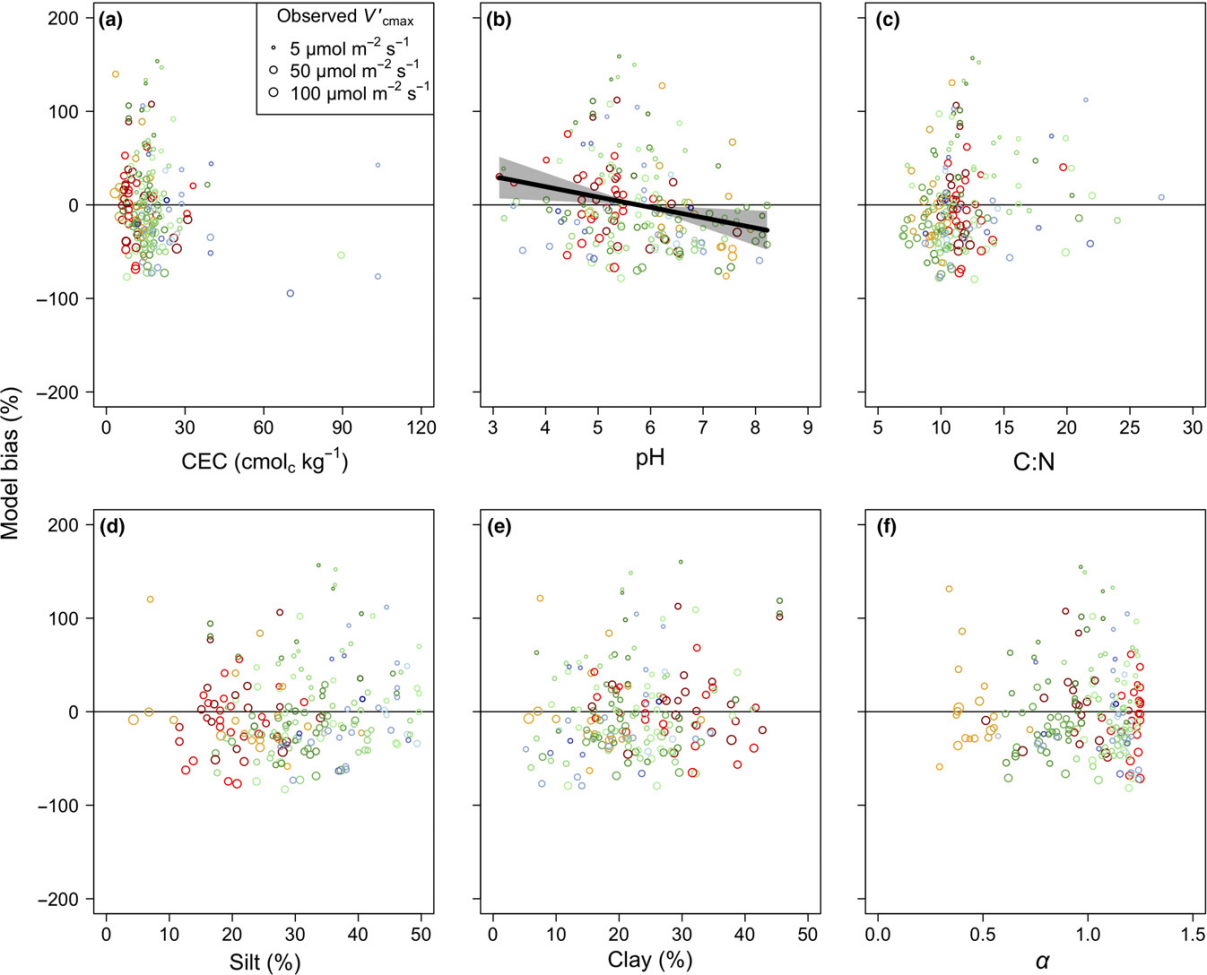
Model bias in relation to soil variables. Partial residuals of the observed bias (%) in the maximum rate of Rubisco carboxylation predicted by the theoretical model ($V_{\mathrm{cmax}}^{\prime }$) by site plotted against soil cation exchange capacity (CEC, panel a), pH (panel b), carbon‐to‐nitrogen ratio (C:N, panel c), silt content (panel d), clay content (panel e), and an index of soil water availability (α; panel f) (black transparent circles). Model bias was defined as $\frac{V_{\mathrm{cmax}\left[\mathrm{pred}\right]}^{\prime }-V_{\mathrm{cmax}\left[\mathrm{obs}\right]}^{\prime }}{V_{\mathrm{cmax}\left[\mathrm{obs}\right]}^{\prime }}*100$, where $V_{\mathrm{cmax}\left[\mathrm{pred}\right]}^{\prime }$ is the predicted optimal $V_{\mathrm{cmax}}^{\prime }$ and $V_{\mathrm{cmax}\left[\mathrm{obs}\right]}^{\prime }$ is the observed $V_{\mathrm{cmax}}^{\prime }$. Data points are sized logarithmically by $V_{\mathrm{cmax}\left[\mathrm{obs}\right]}^{\prime }$. Lines indicate the modelled response from the multiple linear regression models. Shading indicates 95% confidence intervals for regression lines. Only significant trends (*P *<* *0.05) are shown. Data are plotted for each of the 193 sites that had available soil data. Colours are as in Figure 2.

For the 193 sites with soil data, we used a linear model to explore the relative influence of soil nutrient and water supply on bias in our theory. Of six indices of soil nutrient and water availability (soil cation exchange capacity (CEC), soil C:N ratio, soil pH, soil silt content, soil clay content and α), only soil pH had a significant influence (Fig. 5 and Table S4; pH: *F*_1,182_ = 10.14, *P *<* *0.01; all others: *P *>* *0.05). The negative relationship between model bias and pH indicated that our theoretical model tended to overpredict $V_{\mathrm{cmax}\left[\mathrm{obs}\right]}^{\prime }$ as soil acidity increased. To assess the relative influence of climate and soil on $V_{\mathrm{cmax}\left[\mathrm{obs}\right]}^{\prime }$, we quantified the influence of the soil metrics on model predictive ability by comparing three models for predicting $V_{\mathrm{cmax}\left[\mathrm{obs}\right]}^{\prime }$: one based on site climate and elevation (indexed by $V_{\mathrm{cmax}\left[\mathrm{pred}\right]}^{\prime }$), a second model with the six metrics of soil nutrient and water availability only, and a third model based on both climate and soils. The fit of the model that included both $V_{\mathrm{cmax}\left[\mathrm{pred}\right]}^{\prime }$ and soil variables (AIC = 1529.3; *r* ^2^ = 0.68) was slightly better than the model that only included $V_{\mathrm{cmax}\left[\mathrm{pred}\right]}^{\prime }$ (AIC = 1536.4; *r* ^2^ = 0.64) and substantially better than the model that only included the soil variables (AIC = 1669.1; *r* ^2^ = 0.32). These results suggest that soil variables (pH in particular) add statistically significant greater ability to predict $V_{\mathrm{cmax}\left[\mathrm{obs}\right]}^{\prime }$ over biophysical constraints and light availability alone, but that the dominant drivers of $V_{\mathrm{cmax}\left[\mathrm{obs}\right]}^{\prime }$ are captured by our theory.

## Discussion

**Figure 6:**
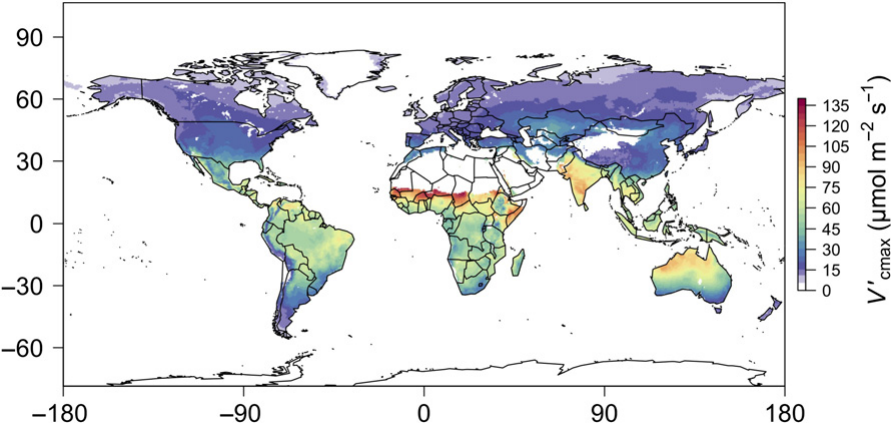
Globally predicted optimal rates of $V_{\mathrm{cmax}}^{\prime }$. Global ‘present‐day’ optimal rates of maximum Rubisco carboxylation ($V_{\mathrm{cmax}}^{\prime }$) computed using mean growing season irradiance, air temperature, vapour pressure deficit and elevation. Values were calculated at 0.5° resolution using effective growing season mean temperature (*T*_g_; °C), atmospheric vapour pressure deficit (*D*_g_; Pa) and incoming photosynthetically active radiation (*I*_g_; μmol m^−2^ s^−1^) for each location from monthly data provided by the Climatic Research Unit (CRU TS3.24.01) (Harris *et al*. 2014). Growing season was defined as months having temperatures greater than 0 °C. Elevation (*z*; m) at each location was obtained from the WFDEI meteorological forcing dataset (Weedon *et al*. 2014). Atmospheric CO_2_ was assumed to be 400 μmol mol^−1^ at *z *=* *0 m and converted to Pa for each location based on *z*.

The broad fidelity of our theory to observations suggests that, across large spatial and phylogenetic scales, realized $V_{\mathrm{cmax}}^{\prime }$ is principally determined by the optimization of photosynthetic processes in response to environmental conditions. Predicted carboxylation capacity is largest in tropical and subtropical regions of the world (Fig. 6), where temperatures and incoming solar radiation are highest. This effect not only follows from the observations presented here (Fig. 2), but also results from temperature (e.g. Smith & Dukes 2017b) and light (e.g. Meir *et al*. 2007) gradient studies. These results suggest that future, warmer conditions may favour increased photosynthetic potential, although this may be balanced by decreases in *V*_cmax_ as a result of elevated CO_2_ (Ainsworth & Rogers 2007).

Nonetheless, there were some significant biases in our model predictions that warrant further discussion. The linear model results indicated a positive bias with light availability, suggesting that the observational data were less sensitive to light availability than predicted by the theory. It is possible that this was driven by individual variation in the realized quantum yield of photosynthetic electron transport (φ), which is the product of the intrinsic quantum efficiency and leaf absorptance of incoming radiation. Previous studies have suggested that intrinsic quantum efficiency and leaf absorptance are not driven by light availability (Evans & Poorter 2001) and, for intrinsic quantum efficiency, that observed variability may be due to measurement technique rather than meaningful biological variation (Skillman 2008). This suggests that the bias in the light response may be due to variability in leaf position and angle, which influence the actual light reaching the leaf surface. Our comparison to the CANTRIP dataset (Keenan & Niinemets 2016) indeed suggests that measured leaves likely were not receiving full sunlight, which would have contributed to the model overestimation that we observed. The combined impact of light availability, leaf position and canopy architecture is a major research need for scaling from leaf to whole‐plant responses at large scales.

Unlike with light availability, there was no bias in our model related to temperature, indicating that the temperature response predicted tends to follow similar responses seen in the global dataset. Notably, the response is also similar to that seen in meta‐analytical (Kattge & Knorr 2007) and controlled‐environment (Scafaro *et al*. 2017; Smith & Dukes 2017b) studies. Nonetheless, temperature was an important determinant of optimal $V_{\mathrm{cmax}}^{\prime }$ rates (Figure 1). Our theory suggests that as temperature increases, higher $V_{\mathrm{cmax}}^{\prime }$ is necessary to support increased electron transport up to their optima. This effect is amplified by a greater stimulation of *K*_c_ compared to the CO_2_ compensation point, Γ*, with temperature (Bernacchi *et al*. 2001). This phenomenon is also observable as a reduction in the optimal ratio of $J_{\max}^{\prime }$ to $V_{\mathrm{cmax}}^{\prime }$ at higher temperatures (Figure S5), an effect consistent with previous studies (e.g. Medlyn *et al*. 2002; Kattge & Knorr 2007; Crous *et al*. 2013; Smith & Dukes 2017b).

It is worth noting that our theory predicts *V*_cmax_ rates at the average growing season temperature (i.e. $V_{\mathrm{cmax}}^{\prime }$), rather than at a standardized temperature. Indeed, *V*_cmax_ at a standardized temperature is likely to be better correlated to *N*_a_ than $V_{\mathrm{cmax}}^{\prime }$ is to *N*_a_ because *V*_cmax_ at a standardized temperature is a proxy for Rubisco content rather than a realized rate. This possibly explains the relatively weaker trend seen here compared to other studies (e.g. Kattge *et al*. 2009; Walker *et al*. 2014). Nonetheless, our strategy allows for a prediction of $V_{\mathrm{cmax}}^{\prime }$ that is as good or better than a recent approach for estimating *V*_cmax_ at a standardized temperature from dynamic allocation of leaf N (Ali *et al*. 2016). Predicting *V*_cmax_ under typical growth conditions is likely more useful for vegetation modelling because it allows for predictions of *V*_cmax_ at temperatures near to the temperatures regularly experienced by plants in a given environment, rather than at a common temperature (e.g. 25 °C), which may be atypical for that environment. Thus, $V_{\mathrm{cmax}}^{\prime }$ would vary temporally owing to comparatively modest diurnal or day‐to‐day temperature variation rather than across large temperature gradients, which will minimize potential predictive errors due to the choice of temperature response functions used to scale $V_{\mathrm{cmax}}^{\prime }$.

Our approach could be extended to examine the influence of temporal variation in environmental conditions on optimal $V_{\mathrm{cmax}}^{\prime }$ predictions. Due to the scale of our analyses and a lack of consistent, high‐resolution environmental data, we used monthly mean data (Harris *et al*. 2014) to create our predictions. While our predictions were able to pick up large spatial trends, the ability of our model to simulate temporal variation is untested here. Better temporal data, coupled with a firmer understanding of the timescale of photosynthetic acclimation, should lead to better temporal predictions.

Our model showed a bias with soil pH, a proxy for soil fertility and leaf *N*_a_. The soil pH effect may be due to the negative effect of soil acidity on nutrient availability, which has been linked to lower rates of photosynthesis (Maire *et al*. 2015). However, because soil acidity tends to correlate with rainfall (Slessarev *et al*. 2016), the overprediction may partly be the result of an overestimation of light availability in wet, tropical regions, as mentioned above. The leaf *N*_a_ effect indicated that the model underestimated $V_{\mathrm{cmax}}^{\prime }$ in high *N*_a_ leaves. This is not surprising, as a substantial amount of leaf *N*_a_ is used for Rubisco (Evans 1989). However, neither soil pH nor leaf *N*_a_, although significant, provided substantial additional explanatory power over climate. By contrast, a substantial portion of global $V_{\mathrm{cmax}}^{\prime }$ is explained by climate alone.

One possible downside to our approach to predicting $V_{\mathrm{cmax}}^{\prime }$ is that our theory, as presented here, does not explicitly include an index of soil moisture and only implements moisture influences through vapour pressure deficit impacts on $C_{i}^{\prime }$. While it is still uncertain how soil moisture influences $V_{\mathrm{cmax}}^{\prime }$ (Smith *et al*. 2014), models that include soil water stress impacts on *V*_cmax_ tend to match observations better than those that do not (Keenan *et al*. 2010). Nonetheless, our model did not show any bias in relation to an index of soil water availability, α. The least‐cost theory, as originally presented (Wright *et al*. 2003), does implicitly assume soil moisture costs to photosynthesis and future work devoted to including these costs explicitly into the quantitative theory could improve model predictions. Optimality based plant hydraulic transport models (e.g. Sperry *et al*. 2017) could be used for this purpose.

Our findings are consistent with the hypothesis that photosynthetic demand drives leaf nitrogen content, rather than the other way around. This was previously suggested by Evans (1989), after which photosynthetic theory has been used to successfully predict leaf nitrogen concentrations (Dong *et al*. 2017). However, most current carbon cycle models utilize leaf N content to predict *V*_cmax_, even those that do not include an interactive N cycle (Smith & Dukes 2013). Our data suggest that leaf N concentration is more likely a consequence of demand for *V*_cmax_. Even so, our theory presents an avenue for reliably predicting $V_{\mathrm{cmax}}^{\prime }$ at global scales without needing to predict *N*_a_, which would reduce model uncertainty.

While we found that collinearity of our data likely had no effect on the results presented here (see VIF analysis in Methods), some degree of collinearity in climate and environmental variables is unavoidable when using natural gradient data. A potential next step in testing our theory is to tailor controlled‐environment studies to assess the individual response of each input of the theoretical model, as well as the influence of soil nutrient availability.

In conclusion, we have developed and tested a theory for predicting environment‐dependent optimal rates of $V_{\mathrm{cmax}}^{\prime }$ against an observational dataset. The agreement between data and theory suggests that plants, through acclimation, adaptation or some combination of the two, are assimilating carbon in an efficient manner by preferentially allocating resources to rate‐limiting processes. This allows for greater resources to be used for non‐photosynthetic processes, such as growth, storage and reproduction, which are important in competitive environments.

## Author contributions

NGS, TFK, ICP and HW designed the study and developed the theoretical model. IJW and ÜN provided input during early stages of the study. NGS performed the analyses. NGS, HW, IJW, ÜN, KYC, TFD, RG, FYI, JK, ELK, VM, AR, SPS, LT, HFT, PAT, MW, LKW and SXZ provided data for the analyses. All authors contributed to the writing of the manuscript.

## Data Availability

Model code can be found at https://github.com/SmithEcophysLab/optimal_vcmax_R (https://doi.org/10.5281/zenodo.1482044). No new data were used in the analyses. Investigators should refer to the citations provided in the Methods section for data access. Please contact Nick Smith (nick.smith@ttu.edu) with any issues.
