# Supplementary material for: Global photosynthetic capacity is optimized to the environment
Source: Ecol Lett. 2019 Jan 4;22(3):506–17. doi: 10.1111/ele.13210 (PMC6849754; doi:10.1111/ele.13210)
Supplement: Supplementary file 1 [file ELE-22-506-s001.docx]

**Title:** Global photosynthetic capacity is optimized to the environment

**Authors:** Nicholas G. Smith^1,2^, Trevor F. Keenan^2,3^, I. Colin Prentice^4,5,6^, Han Wang^5,7^, Ian J. Wright^6^, Ülo Niinemets^8^, Kristine Y. Crous^9^, Tomas F. Domingues^10^, Rossella Guerrieri^11,12^, F. Yoko Ishida^13^, Jens Kattge^14,15^, Eric L. Kruger^16^, Vincent Maire^17^, Alistair Rogers^18^, Shawn P. Serbin^18^, Lasse Tarvainen^19^, Henrique F. Togashi^6^, Philip A. Townsend^16^, Meng Wang^5,20^, Lasantha K. Weerasinghe^21,22^, Shuang-Xi Zhou^6,23^

^1^Department of Biological Sciences, Texas Tech University, Lubbock, TX, USA

^2^Climate and Ecosystem Sciences Division, Lawrence Berkeley National Laboratory, Berkeley, CA, USA

^3^Department of Environmental Science, Policy and Management, UC Berkeley, Berkeley, CA, USA

^4^AXA Chair of Biosphere and Climate Impacts, Department of Life Sciences, Imperial College London, London, UK

^5^College of Forestry, Northwest A & F University, Yangling, China

^6^Department of Biological Sciences, Macquarie University, NSW 2109, Australia

^7^Department of Earth System Science, Tsinghua University, Beijing

^8^Department of Plant Physiology, Institute of Agricultural and Environmental Sciences, Estonian University of Life Sciences, Tartu, Estonia

^9^Hawkesbury Institute for the Environment, Western Sydney University, Penrith, Australia

^10^Departamento de Biologia, Faculdade de Filosofia, Ciências e Letras de Ribeirão Preto - University of São Paulo, São Paulo, Brazil

^11^Center for Ecological Research and Forestry Applications, Universidad Autonoma de Barcelona, Cerdanyola, Barcelona, Spain

^12^School of Geosciences, University of Edinburgh, Edinburgh, UK

^13^Centre for Tropical Environmental and Sustainability Science, College of Science and Engineering, James Cook University, Cairns, Australia

^14^Max Planck Institute for Biogeochemistry, Jena, Germany

^15^German Center for Integrative Biodiversity Research Halle-Jena-Leipzig, Leipzig, Germany

^16^Department of Forest and Wildlife Ecology, University of Wisconsin – Madison, Madison, Wisconsin, USA

^17^Département des sciences de l’environnement, Université du Québec à Trois, Rivières, Trois Rivières, Canada

^18^Environmental and Climate Sciences Department, Brookhaven National Laboratory, Upton, NY, USA

^19^Department of Biological and Environmental Sciences, University of Gothenburg, Gothenburg, Sweden

^20^ State Environmental Protection Key Laboratory of Wetland Ecology and Vegetation Restoration, Northeast Normal University, Changchun, China

^21^Research School of Biology, The Australian National University, Canberra, Australia

^22^Faculty of Agriculture, University of Peradeniya, Peradeniya, Sri Lanka

^23^The New Zealand Institute for Plant and Food Research Ltd, Hawke’s Bay, New Zealand

**Supplementary Text**

*Calculation of non-varying parameter c in optimal V_cmax_ model*

The parameter *c* in equation 14 was assumed to be non-varying and, thus estimated under “standard conditions” defined as a temperature of 25°C, sea level atmospheric pressure, 1 kPa vapor pressure deficit, and 360 ppm CO_2_. We calculated *c* by rearranging equations 15 and 19 and solving for the ratio of *J_max_* to *V_cmax_* (*r_jv_)*:

$r_{jv}=\frac{J_{max}}{V_{cmax}}=\frac{8\theta m_{c}\varpi}{{m\varpi}^{*}}$ (S1)

We then solve for $\varpi$ in relation to *r_jv_*. Rearranging that equation, we define a new variable (*v*), which is a function of $\varpi$.

$v=\frac{\varpi}{\varpi^{*}}$ (S2)

where

$v=\frac{r_{jv}m}{8\theta m_{c}}$ (S3)

The $\varpi^{*}$ term can then be expanded and $\varpi$ represented as a function of $v$ using the non-zero root

$\varpi=\frac{2-4\theta v}{\frac{1}{v}-2}$ (S4)

This alternate representation of $\varpi$ can be used to solve for *c* as

$-\theta\left( \frac{4c}{m} \right)^{2}+\left( \frac{4c}{m} \right)-\frac{1}{\left( \frac{\left( \varpi+\left( 1-2\theta\right) \right)^{2}}{1-\theta} \right)+4\theta}=0$ (S5)

Under standard conditions we take *r_jv_*= 2.07 (Smith & Dukes 2017), *c* is then equal to 0.053. This value can then be used to compute $\varpi$ in equation 16 in the main text.

*Potential common element correlation in temperature scaling of V_cmax_*

To aid in comparison of the observed to modeled $V_{\mathrm{cmax}}^{'}$ (i.e., Figure 2 of the main text), both the modeled and observed data were scaled to *T­*_g_ using a similar function (Eqns. 20 and 22). The two functions differed in the temperature from which the values were scaled, but nonetheless had similar shapes. While it was necessary to apply the function, the similar shape introduced the potential for a spurious correlation between the modeled and observed values because of the common element applied (i.e., similar temperature response) to each value (Chayes 1971).

We examined the influence of the common temperature response function using two approaches, similar to the techniques employed by Lloyd *et al.* (2013) for examining spurious correlations in leaf trait data. First, we calculated a null regression coefficient (*ρ*) (Chayes 1971) for our data using Eqn. 6.2 in Lloyd *et al.* (2013) and restructured it for non-log transformed data:

$\rho=\frac{\mu_{Tobs}\sigma_{Tobs}}{\sqrt{\mu_{Tobs}^{2}\sigma_{Tobs}^{2}+\mu_{Vobs}^{2}\sigma_{Vobs}^{2}}}\frac{\mu_{Tmod}\sigma_{Tmod}}{\sqrt{\mu_{Tmod}^{2}\sigma_{Tmod}^{2}+\mu_{Vmod}^{2}\sigma_{Vmod}^{2}}}$ (S7)

where $\mu_{Tobs}$ and $\sigma_{Tobs}$ are the mean and standard deviation of the temperature response scalar applied to the observed $V_{\mathrm{cmax}}^{'}$, respectively, $\mu_{Vobs}$ and $\sigma_{Vobs}$ are the mean and standard deviation of the unscaled observed $V_{\mathrm{cmax}}^{'}$, respectively, $\mu_{Tmod}$ and $\sigma_{Tmod}$ are the mean and standard deviation of the temperature response scalar applied to the modeled $V_{\mathrm{cmax}}^{'}$, respectively, $\mu_{Vmod}$ and $\sigma_{Vmod}$ are the mean and standard deviation of the unscaled modeled $V_{\mathrm{cmax}}^{'}$, respectively. Using Eqn. S7, we calculated *ρ* for the site-level dataset (n=201) as <1*e*-9, indicating a negligible null correlation between the data and, thus, little evidence for a spurious correlation.

Second, we employed a randomization technique to search for evidence of a spurious correlation. We randomly sampled 201 unscaled observed and modeled $V_{\mathrm{cmax}}^{'}$ values from normally distributed values defined by the mean and standard deviations of the unscaled observed and modeled $V_{\mathrm{cmax}}^{'}$ values, respectively. We then applied temperature scalars used in the analyses, paired for each observed and modeled value, to the randomized data. Next, we calculated the correlation between the unscaled random observed and modeled $V_{\mathrm{cmax}}^{'}$ data as well as the correlation between the scaled random observed and modeled $V_{\mathrm{cmax}}^{'}$ data. We repeated this process 10,000 times to generate a distribution of correlations. The center of the distribution for the unscaled data fell between r^2^ values of 0 and 0.01 (mean = 0.005; Figure S6). By comparison, the center of the distribution for the scaled data fell between r^2^ values of 0.08 and 0.09 (mean = 0.087; Figure S6). This suggests that the similar temperature scalar did result in some degree of spurious correlation. However, the correlation in the random data was less than 1/7^th^ of the correlation between the modeled and observed data in our study (r^2^=0.64), indicating a low degree of spurious correlation presented in the results of the main text.

Taken together, these results indicate that the necessary temperature response scaling performed during our analyses did not result in a spurious correlation and that the results would hold under conditions where temperature response scaling was not necessary (e.g., data measured at the temperature to which the plant is acclimated).

*On the ratio of* $J_{\max}^{'}$ *to* $V_{\mathrm{cmax}}^{'}$

The optimal ratio of *J*_max_ to *V*_cmax_ (eqn. S1) is independent of light, but decreases with increasing temperature, vapor pressure deficit, and elevation (Figure S5).

*Model-data comparison with only A_net_-C_i_ curve derived data*

Some of the *V*_cmax_ data from the *GlobV* dataset were derived using the one-point method (method presented in De Kauwe *et al*. (2015)). Analyses have shown that *V*_cmax_ derived using the one-point method is similar to *V*_cmax_ derived from net photosynthesis (*A*_net_)-intercellular CO_2_ (*C*_i_) response (*A-C*_i_) curves (De Kauwe *et al.* 2015). However, some conditions and measurement protocols may cause bias in one-point estimates. First, conditions that result in low stomatal conductance may result in a low signal-to-noise ratio for estimated *A*_net_ and *C*_i_ values. Additionally, parameters estimated using temperature, including respiration, CO_2_ compensation points, and Michaelis-Menten coefficients could be biased due to parameter uncertainty in the temperature response functions. Respiration, even if measured, may still bias estimates because it is typically a much smaller flux than photosynthesis. Finally, assuming a CO_2_ transition point at which *A*_net_ switches from being limited by Rubisco to being limited by electron transport may be problematic if this point varies by species or environmental condition (De Kauwe *et al.* 2015).

To assess whether our results were biased as a result of using one-point-derived data, we ran a model-data comparison using only data derived from *A-C*_i_ response curves. We removed data that we were sure came from one-point measurements as well as data for which we did not know how *V*_cmax_ was derived. The removed data included those from Maire *et al.* (2015), Niinemets *et al.* (2015), Keenan & Niinemets (2016), Wang et al. (2017a), and the TRY plant trait database (Kattge *et al.* 2011). After removal, 2059 data points at 55 0.5°x0.5° sites remained. When compared with the *A*_net_-*C*_i_ response curve-derived data only, our theoretical model was able to capture 69% of the total variation in observed $V_{\mathrm{cmax}}^{'}$ values (Figure S7). These results indicate that the fidelity of our model to the data was not due to the way in which V_cmax_ was derived.

**Figure S1.**

**
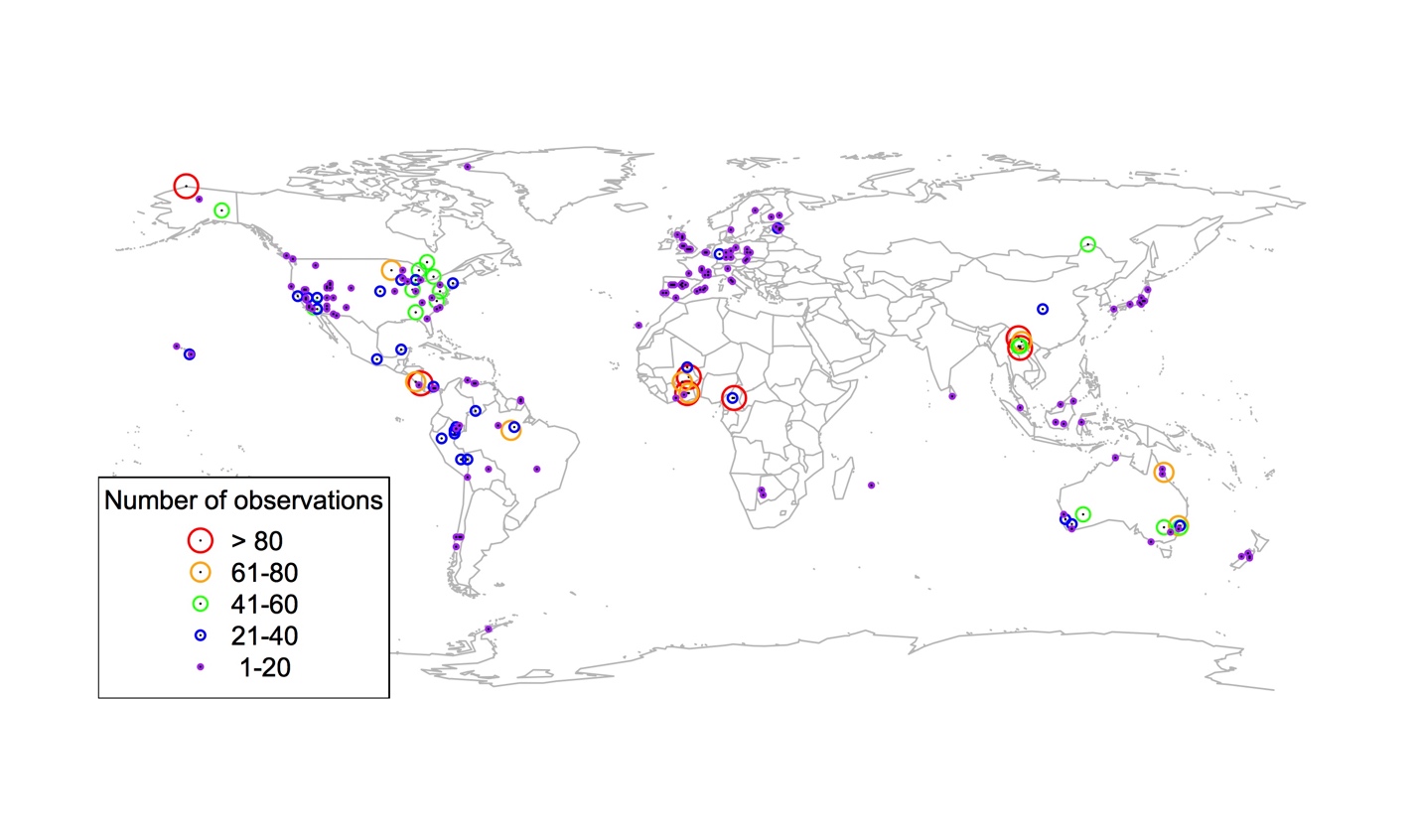
**

Figure S1. Map of data included in the GlobV dataset. Sites are binned by the number of observations at each site. Sites with >80, 61-80, 41-60, 21-40, and <20 observations are denoted by red, orange, green, blue, and purple points, respectively, with decreasing size.

**Figure S2.**

**
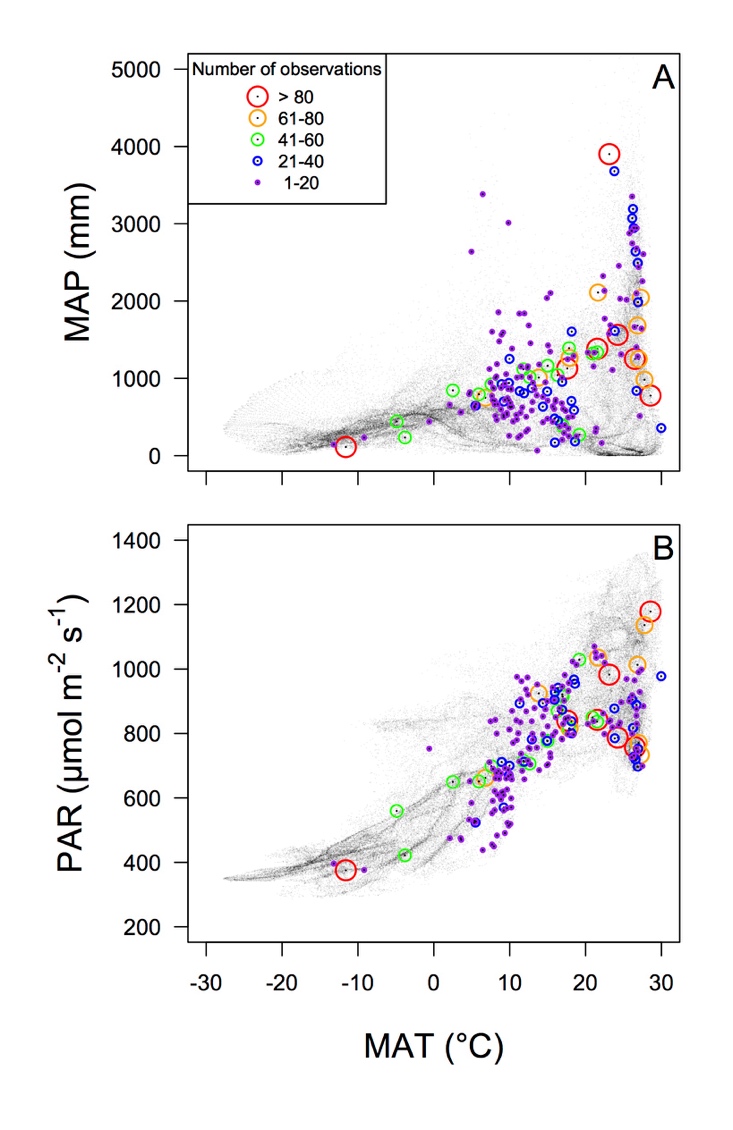
**

Figure S2. (Top) Mean annual precipitation (MAP; mm) and (bottom) photosynthetically active radiation (PAR; µmol m^-2^ s^-1^) plotted against mean annual temperature (MAT) from 1901-2015 for sites in the observational dataset from 0.5° resolution data provided by the Climatic Research Unit (CRU TS3.24.01)(Harris *et al.* 2014). Colors and sizes are as in Figure S1. Small grey dots indicate data for all land surface grid cells on Earth. Note that growing season, not mean annual, data are used for the analyses presented in the text. These data are presented to show the climatic extent of the *GlobV* dataset.

**Figure S3.**


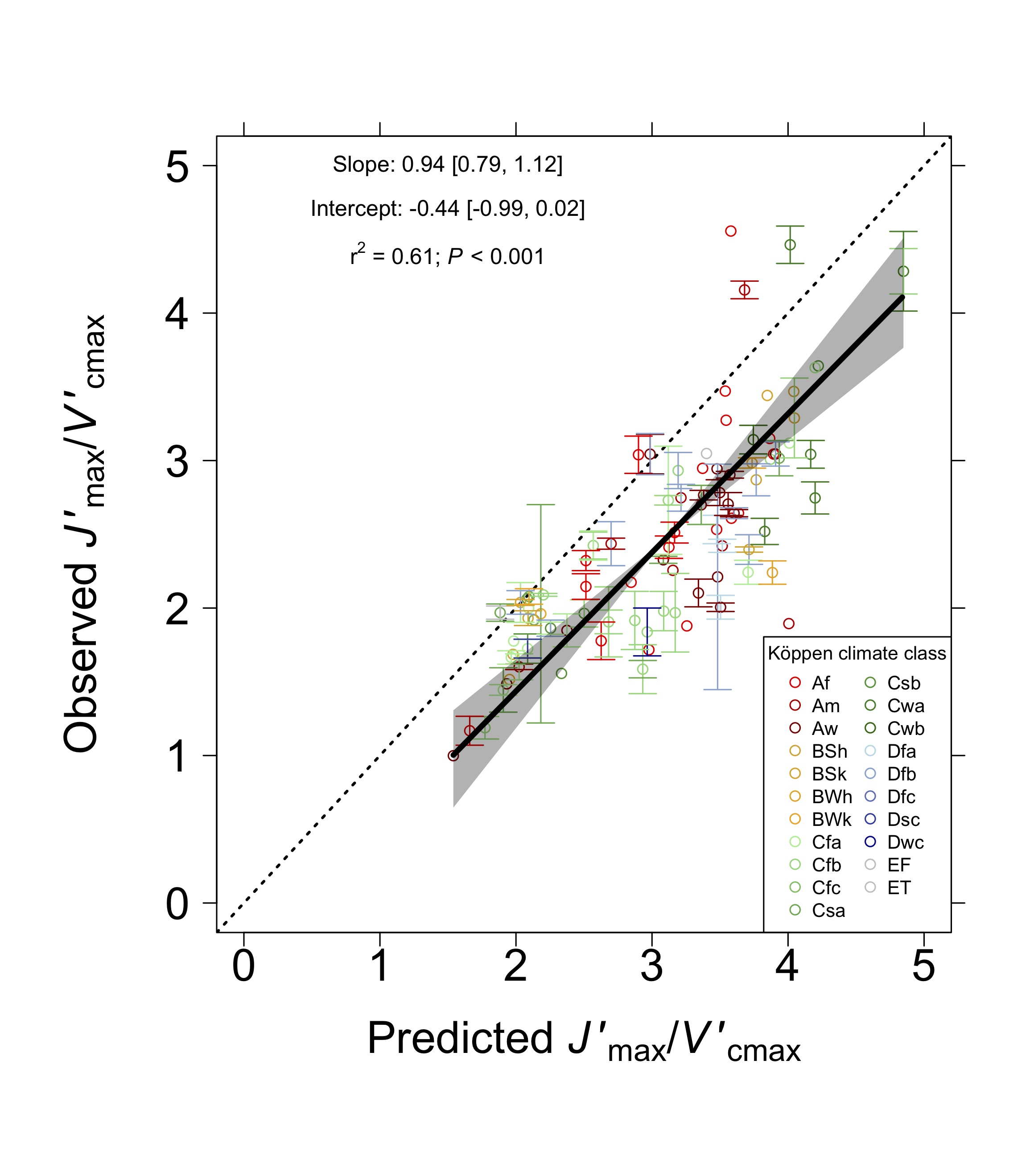


**Figure S3. Comparison of observed to optimal** $\boldsymbol{J}_{\mathbf{max}}^{\boldsymbol{'}}$**/**$\boldsymbol{V}_{\mathbf{cmax}}^{\boldsymbol{'}}$**.** Observed mean ratio of the maximum rate of electron transport ($J_{\max}^{'}$) to the maximum rate of Rubisco carboxylation ($V_{\mathrm{cmax}}^{'}$) at 90 global sites plotted against the predicted $J_{\max}^{'}$/$V_{\mathrm{cmax}}^{'}$value at that site from the theoretical model. Note that values were standardized to T_g_ (see Materials and Methods). Sites are colored by Köppen climate classification. Tropical (first letter A), arid (first letter B), temperate (first letter C), boreal (first letter D), and polar (first letter E) regions are represented by red, yellow, green, blue, and grey colors. Error bars represent standard errors of the mean. The solid black line is the best fit line from the reduced major axis regression. The grey shaded area represents a 95% confidence interval. The dotted black line is a 1:1 line. Köppen climate classification key: Af= tropical rainforest, Am= tropical monsoon, Aw= tropical wet savannah, BSh= hot arid steppe, BSk= cold arid steppe, BWh= hot arid desert, BWk= cold arid desert, Cfa= temperate hot summer without dry season, Cfb= temperate warm summer without dry season, Cfc= temperate cold summer without dry season, Csa= temperate hot summer with dry summer, Csb= temperate warm summer with dry summer, Cwa= temperate hot summer with dry winter, Cwb= temperate warm summer with dry winter, Dfa= boreal hot summer without dry season, Dfb= boreal warm summer without dry season, Dfc= boreal cold summer without dry season, Dsc= boreal cold summer with dry summer, Dwc= boreal cold summer with dry winter, EF= eternal winter, ET= tundra.

**Figure S4.**

**
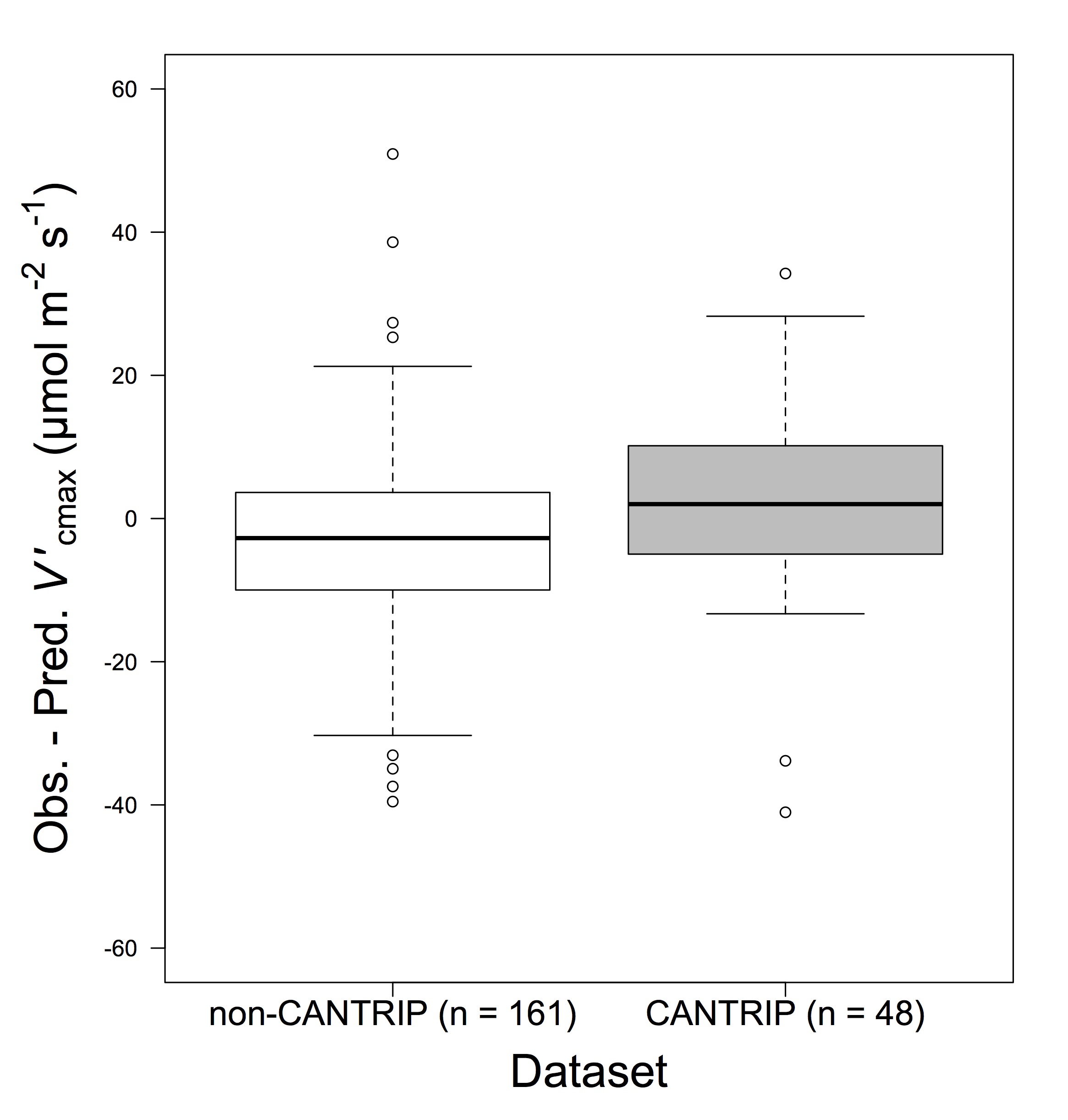
**

Figure S4. The difference between observed and predicted $V_{\mathrm{cmax}}^{'}$ values using the non-CANTRIP $V_{\mathrm{cmax}}^{'}$ values (white) and high light (*Q_int_* = 40 mol m^-2^ d^-1^) CANTRIP (Keenan & Niinemets 2016) values (grey).

**Figure S5.**

**
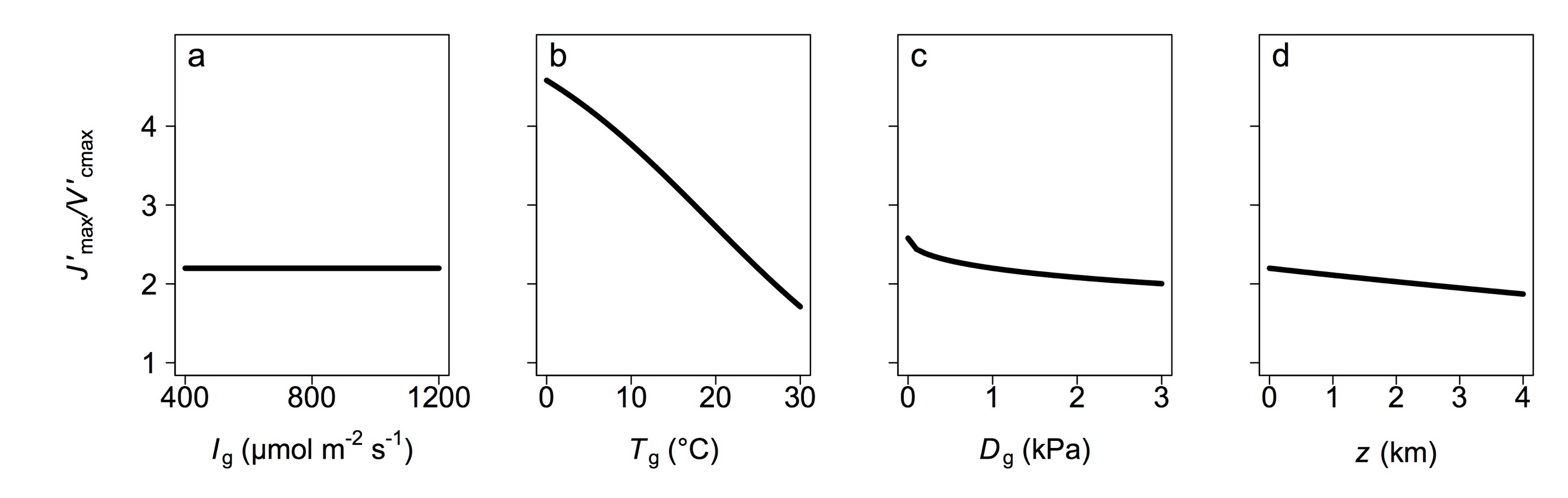
**

Figure S5. Theoretical ratio of the maximum rate of electron transport ($J_{\max}^{'}$) to the maximum rate of Rubisco carboxylation ($V_{\mathrm{cmax}}^{'}$) sensitivity to the main environmental parameters within the model: growing season mean irradiance (I_g_; µmol m^-2^ s^-1^), growing season mean temperature (T_g_; °C), growing season mean vapor pressure deficit (D_g_; kPa), and elevation (z; m). Sensitivity analyses were done while keeping all other environmental variables at standard values as in Figure 1: I_g_ = 800 µmol m^-2^ s^-1^, T_g_ = 25°C, D_g_ = 1 kPa, z = 0 km.

**Figure S6.**

**
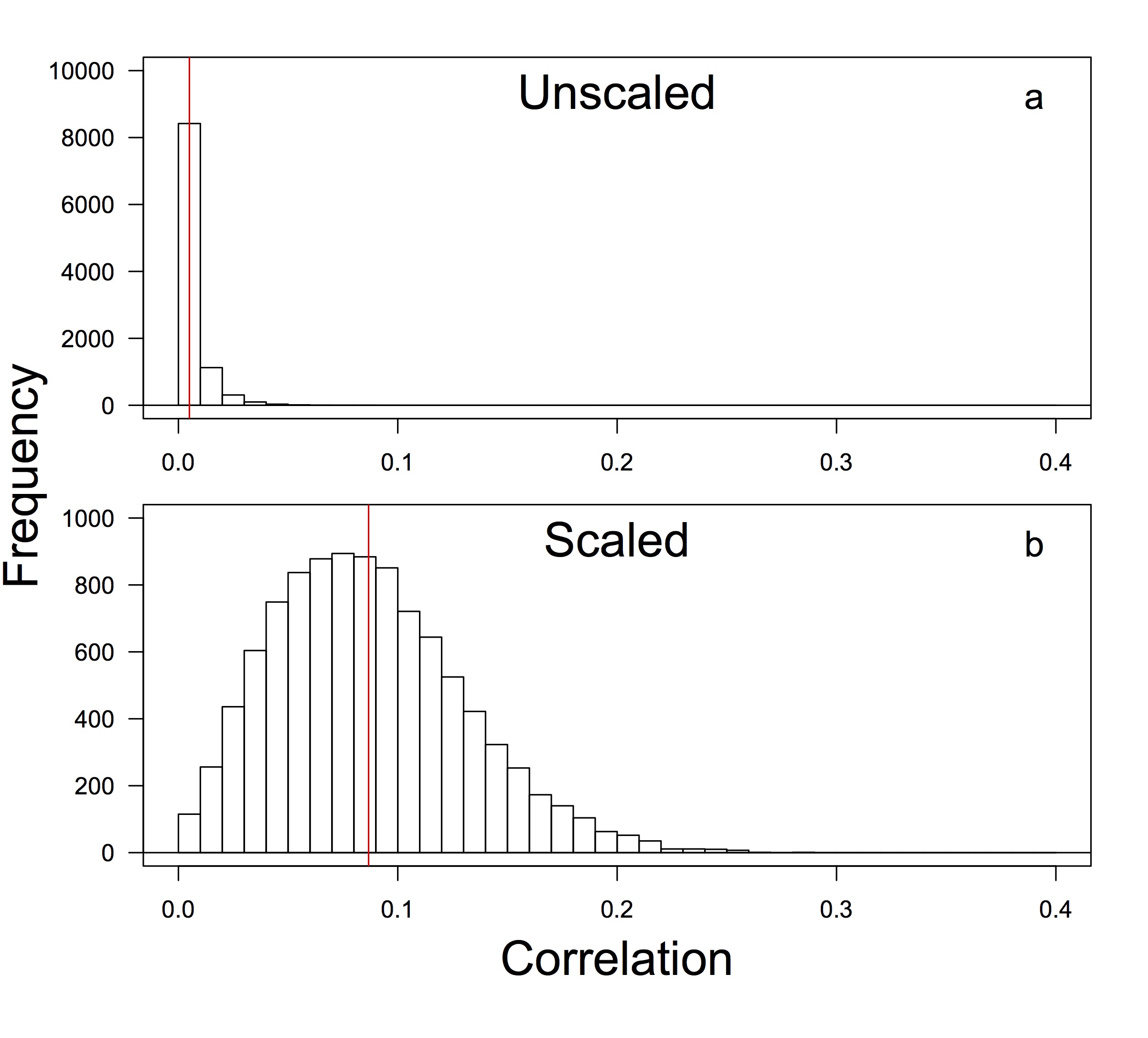
**

Figure S6. The correlation (r^2^) between 10,000 randomly generated populations of observed and modeled $V_{\mathrm{cmax}}^{'}$ data. The top panel shows the correlation between observed and modeled $V_{\mathrm{cmax}}^{'}$ within each population. The bottom panel shows the same correlation following the application of non-random sets of temperature response scalars. Red lines indicate location of the mean across all populations within each panel. Random observed and modeled $V_{\mathrm{cmax}}^{'}$ data were generated by randomly sampling 201 unscaled observed and modeled $V_{\mathrm{cmax}}^{'}$ values from normally distributed values defined by the mean and standard deviations of the unscaled observed and modeled $V_{\mathrm{cmax}}^{'}$ values, respectively. This was done 10,000 times, creating the 10,000 populations present in the histogram.

**Figure S7**


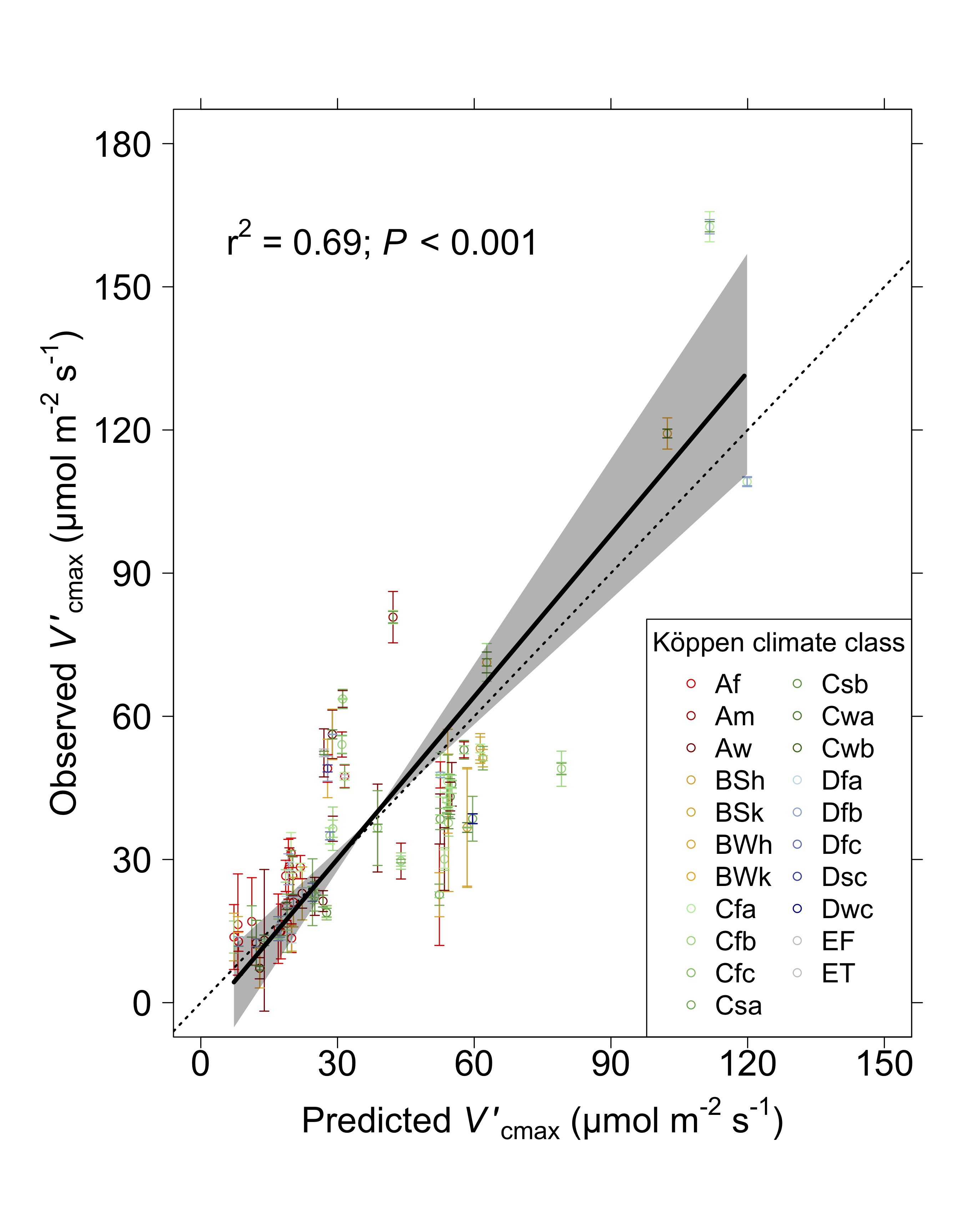


**Figure S7. Comparison of observed to optimal** $\boldsymbol{V}_{\mathbf{cmax}}^{\boldsymbol{'}}$ **using only data derived from net photosynthesis (*A*_net_)-intercellular CO_2_ (*C*_i_) response curves.** Observed mean maximum rate of Rubisco carboxylation ($V_{\mathrm{cmax}}^{'}$) at 55 global sites plotted against the predicted $V_{cmax}^{'}$value at that site from the theoretical model. Sites are colored by Köppen climate classification. Tropical (first letter A), arid (first letter B), temperate (first letter C), boreal (first letter D), and polar (first letter E) regions are represented by red, yellow, green, blue, and grey colors. Error bars represent standard errors of the mean. The solid black line is the best fit line from the reduced major axis regression. The grey shaded area represents a 95% confidence interval. The dotted black line is a 1:1 line. Köppen climate classification key: Af= tropical rainforest, Am= tropical monsoon, Aw= tropical wet savannah, BSh= hot arid steppe, BSk= cold arid steppe, BWh= hot arid desert, BWk= cold arid desert, Cfa= temperate hot summer without dry season, Cfb= temperate warm summer without dry season, Cfc= temperate cold summer without dry season, Csa= temperate hot summer with dry summer, Csb= temperate warm summer with dry summer, Cwa= temperate hot summer with dry winter, Cwb= temperate warm summer with dry winter, Dfa= boreal hot summer without dry season, Dfb= boreal warm summer without dry season, Dfc= boreal cold summer without dry season, Dsc= boreal cold summer with dry summer, Dwc= boreal cold summer with dry winter, EF= eternal winter, ET= tundra.

**Figure S8**

**Figure S8. Comparison of observed to optimal** $\boldsymbol{V}_{\mathbf{cmax}}^{\boldsymbol{'}}$ **for each individual datapoint.** Observed mean maximum rate of Rubisco carboxylation ($V_{\mathrm{cmax}}^{'}$) plotted against the predicted $V_{cmax}^{'}$value from the theoretical model for each individual GlobV datapoint. Points are colored by Köppen climate classification. Tropical (first letter A), arid (first letter B), temperate (first letter C), boreal (first letter D), and polar (first letter E) regions are represented by red, yellow, green, blue, and grey colors. The solid black line is the best fit line from the reduced major axis regression. The grey shaded area represents a 95% confidence interval. The dotted black line is a 1:1 line. Köppen climate classification key: Af= tropical rainforest, Am= tropical monsoon, Aw= tropical wet savannah, BSh= hot arid steppe, BSk= cold arid steppe, BWh= hot arid desert, BWk= cold arid desert, Cfa= temperate hot summer without dry season, Cfb= temperate warm summer without dry season, Cfc= temperate cold summer without dry season, Csa= temperate hot summer with dry summer, Csb= temperate warm summer with dry summer, Cwa= temperate hot summer with dry winter, Cwb= temperate warm summer with dry winter, Dfa= boreal hot summer without dry season, Dfb= boreal warm summer without dry season, Dfc= boreal cold summer without dry season, Dsc= boreal cold summer with dry summer, Dwc= boreal cold summer with dry winter, EF= eternal winter, ET= tundra.

**Table S1. Bias in the optimal** $\boldsymbol{V}_{\mathbf{cmax}}^{\boldsymbol{'}}$ **prediction by environmental variables (full model r^2^ = 0.14)**

| Predictor | Df | Sum Sq | Mean Sq | *F* | *P* | *VIF* |
| --- | --- | --- | --- | --- | --- | --- |
| *I_g_* | 1 | 27109.47 | 27109.47 | 11.54 | 0.001 | 2.37 |
| *T_g_* | 1 | 5152.61 | 5152.61 | 2.19 | 0.140 | 1.85 |
| *D_g_* | 1 | 18003.81 | 18003.81 | 7.66 | 0.006 | 2.59 |
| *z* | 1 | 27300.70 | 27300.70 | 11.62 | 0.001 | 1.59 |
| Residuals | 196 | 460434.81 | 2349.16 |  |  |  |

Key: Df = degrees of freedom, Sum Sq = sum of squares, Mean Sq = mean squares, *F* = F statistic, *P* = *P* statistic, *VIF* = variance inflation factor

**Table S2. Bias in the optimal** $\boldsymbol{V}_{\mathbf{cmax}}^{\boldsymbol{'}}$ **prediction by environmental variables and leaf nitrogen per area (*N*_a_) (full model r^2^ = 0.36)**

| Predictor | Df | Sum Sq | Mean Sq | *F* | *P* | *VIF* |
| --- | --- | --- | --- | --- | --- | --- |
| *I_g_* | 1 | 9009.58 | 9009.58 | 4.47 | 0.037 | 1.94 |
| *T_g_* | 1 | 1866.51 | 1866.51 | 0.93 | 0.339 | 2.22 |
| *D_g_* | 1 | 703.76 | 703.76 | 0.35 | 0.556 | 2.96 |
| *z* | 1 | 35210.36 | 35210.36 | 17.46 | <0.001 | 1.80 |
| *N_a_* | 1 | 59851.38 | 59851.38 | 29.67 | <0.001 | 1.16 |
| Residuals | 92 | 185582.56 | 2017.20 |  |  |  |

Key: Df = degrees of freedom, Sum Sq = sum of squares, Mean Sq = mean squares, *F* = F statistic, *P* = *P* statistic, *VIF* = variance inflation factor

**Table S3. Bias in the optimal** $\boldsymbol{V}_{\mathbf{cmax}}^{\boldsymbol{'}}$ **prediction by environmental variables and leaf mass per area (*LMA*) (full model r^2^ = 0.19)**

| Predictor | Df | Sum Sq | Mean Sq | *F* | *P* | *VIF* |
| --- | --- | --- | --- | --- | --- | --- |
| *I_g_* | 1 | 18197.41 | 18197.41 | 7.01 | 0.009 | 2.15 |
| *T_g_* | 1 | 6111.68 | 6111.68 | 2.36 | 0.128 | 2.24 |
| *D_g_* | 1 | 1073.91 | 1073.91 | 0.41 | 0.521 | 2.96 |
| *z* | 1 | 39875.60 | 39875.60 | 15.37 | <0.001 | 1.86 |
| *LMA* | 1 | 242.11 | 242.11 | 0.09 | 0.761 | 1.18 |
| Residuals | 106 | 275029.79 | 2594.62 |  |  |  |

Key: Df = degrees of freedom, Sum Sq = sum of squares, Mean Sq = mean squares, *F* = F statistic, *P* = *P* statistic, *VIF* = variance inflation factor

**Table S4. Bias in the optimal** $\boldsymbol{V}_{\mathbf{cmax}}^{\boldsymbol{'}}$ **prediction by environmental variables and soil variables (full model r^2^ = 0.20)**

| Predictor | Df | Sum Sq | Mean Sq | *F* | *P* | *VIF* |
| --- | --- | --- | --- | --- | --- | --- |
| *I_g_* | 1 | 21204.94 | 21204.94 | 9.32 | 0.003 | 3.08 |
| *T_g_* | 1 | 3714.59 | 3714.59 | 1.63 | 0.203 | 4.54 |
| *D_g_* | 1 | 15172.18 | 15172.18 | 6.67 | 0.011 | 3.71 |
| *z* | 1 | 27042.81 | 27042.81 | 11.89 | 0.001 | 1.82 |
| CEC | 1 | 85.67 | 85.67 | 0.04 | 0.846 | 2.25 |
| pH | 1 | 23074.54 | 23074.54 | 10.14 | 0.002 | 1.85 |
| CN | 1 | 2698.53 | 2698.53 | 1.19 | 0.278 | 2.29 |
| Silt % | 1 | 2937.22 | 2937.22 | 1.29 | 0.257 | 3.01 |
| Clay % | 1 | 5538.24 | 5538.24 | 2.43 | 0.120 | 1.50 |
| *α* | 1 | 10.12 | 10.12 | <0.01 | 0.947 | 4.00 |
| Residuals | 182 | 414056.66 | 2275.04 |  |  |  |

Key: Df = degrees of freedom, Sum Sq = sum of squares, Mean Sq = mean squares, *F* = F statistic, *P* = *P* statistic, *VIF* = variance inflation factor

**References**

1.Chayes, F. (1971). *Ratio correlation: a manual for students of petrology and geochemistry*. University of Chicago Press, Chicago and London

2.Harris, I., Jones, P.D., Osborn, T.J. & Lister, D.H. (2014). Updated high-resolution grids of monthly climatic observations – the CRU TS3.10 Dataset. *Int. J. Climatol.*, 34, 623–642

3.Kattge, J., Diaz, S., Lavorel, S., Prentice, I.C., Leadley, P., Bönisch, G., *et al.* (2011). TRY–a global database of plant traits. *Glob. Chang. Biol.*, 17, 2905–2935

4.De Kauwe, M.G., Lin, Y., Wright, I.J., Medlyn, B.E., Crous, K.Y., Ellsworth, D.S., *et al.* (2015). A test of the ‘one‐point method’for estimating maximum carboxylation capacity from field‐measured, light‐saturated photosynthesis. *New Phytol.*, 210, 1130–1144

5.Keenan, T.F. & Niinemets, Ü. (2016). Global leaf trait estimates biased due to plasticity in the shade. *Nat. Plants*, 3, 16201

6.Lloyd, J., Bloomfield, K., Domingues, T.F. & Farquhar, G.D. (2013). Photosynthetically relevant foliar traits correlating better on a mass vs an area basis: of ecophysiological relevance or just a case of mathematical imperatives and statistical quicksand? *New Phytol.*, 199, 311–321

7.Maire, V., Wright, I.J., Prentice, I.C., Batjes, N.H., Bhaskar, R., Bodegom, P.M., *et al.* (2015). Global effects of soil and climate on leaf photosynthetic traits and rates. *Glob. Ecol. Biogeogr.*, 24, 706–717

8.Niinemets, Ü., Keenan, T.F. & Hallik, L. (2015). A worldwide analysis of within-canopy variations in leaf structural, chemical and physiological traits across plant functional types. *New Phytol.*, 205, 973–993

9.Smith, N.G. & Dukes, J.S. (2017). Short-term acclimation to warmer temperatures accelerates leaf carbon exchange processes across plant types. *Glob. Chang. Biol.*, 23, 4840–4853

10.Wang, H., Harrison, S., Prentice, I., Yang, Y., Bai, F., Togashi, H., *et al.* (2017). The China Plant Trait Database: towards a comprehensive regional compilation of functional traits for land plants. *Ecology*, 99, 500–500
